# Biomedical Applications and Nutritional Value of Specific Food-Derived Polysaccharide-Based Hydrogels

**DOI:** 10.1016/j.advnut.2024.100309

**Published:** 2024-09-28

**Authors:** Qianru Xiang, Yuting Hao, Zijun Xia, Meiqi Liao, Xinkai Rao, Shenghui Lao, Qi He, Congshun Ma, Wenzhen Liao

**Affiliations:** 1 Department of Nutrition and Food Hygiene, Guangdong Provincial Key Laboratory of Tropical, School of Public Health, Southern Medical University, Guangzhou, China; 2 National Health Commission (NHC) Key Laboratory of Male Reproduction and Genetics, Guangzhou, China; 3 Department of Reproductive Medicine Center, Guangdong Provincial Reproductive Science Institute (Guangdong Provincial Fertility Hospital), Guangzhou, China; 4 Hospital Infection Control Office, Guangzhou Elderly Hospital, Guangzhou, China; 5 School of Basic Medical Sciences, Southern Medical University, Guangzhou, China; 6 Disease Research, First clinical medical College, Southern Medical University, Guangzhou, China; 7 Department of Critical Care Medicine, First Affiliated Hospital of Sun Yat sen University, Guangzhou, China; 8 School of Public Health, Southern Medical University, Guangzhou, China

**Keywords:** hydrogel, polysaccharide, nutritional value, biomedical applications, food source

## Abstract

Food-derived polysaccharide-based hydrogels (FPBHs), which are composed of polysaccharides derived from food sources exhibit great potential for biomedical applications. The FPBHs possess a wide range of biological activities and can be utilized in the treatment of various clinical diseases. However, the majority of research efforts have predominantly focused on nonspecific polysaccharides derived from various sources (most plants, animals, and microorganisms), whereas the exploration of hydrogels originating from specific polysaccharides with distinct bioactivity extracted from natural food sources remains limited. In this review, a comprehensive search was conducted across 3 major databases (PubMed, Web of Science, and Medline) until October 24, 2024 to include 32 studies that employed FPBHs for biomedical applications. This review provides an overview of hydrogels based on specific food-derived polysaccharides by summarizing their types, sources, molecular weight, monosaccharide composition, and biological activities. The crosslinking strategies employed in the fabrication of FPBHs were demonstrated. The attributes and characteristics of FPBHs were delined, including their physical, chemical, and functional properties. Of particular note, the review highlights *in vivo* and *in vitro* studies exploring the biomedical applications of FPBHs and delve into the nutritional value of specific food-derived polysaccharides. The challenges encountered in basic research involving FPBHs were enumerated as well as limitation in their clinical practice. Finally, the potential market outlook for FPBHs in the future was also discussed.

**Statement of Significance**

Food-derived polysaccharide-based hydrogels exhibit a wide range of biological activities and can be utilized in the treatment of various clinical diseases. However, the majority of research efforts have predominantly focused on nonspecific polysaccharides derived from various sources (most plants, animals, and microorganisms), although the exploration of hydrogels originating from specific polysaccharides with distinct bioactivity extracted from natural food sources remains limited.

## Introduction

In the realm of biomedical sciences, gels are typically characterized by their high degree of hydration, and consist of 2 or more components in varying proportions, namely a heavier mass solvent and either natural or synthetic macromolecular polymer solutes [1]. Based on their physical properties, gels are generally categorized into hydrogels, oleogels (or organogels/bigels), aerogels, cryogels, and xerogels [2]. Of these, hydrogels have garnered substantial attention in various fields, including environmental engineering, biomedicine, and biotechnology. Among these categories, the hydrophilic properties and potential biocompatibility of the initially synthesized crosslinked hydrogels garnered significant attention from biomaterials experts in 1960 [3], leading to extensive research endeavors to optimize their performance.

Although there is debate about their precise definition, most researchers refer to hydrogels as collections of hydrophilic molecules and one or more composite biopolymers with water as the main phase [2]. Hydrogels swell when exposed to water and can hold a large amount of water within their volume. Compared to other types of gels, hydrogels exhibit superior characteristics such as structural stability, high absorbency, strong permeability, self-healing, adhesion, and versatile stimulus response [4]. Polymers can be classified into 2 categories, synthetic polymers and natural polymers. Similarly, hydrogels composed of different types of polymers can be further categorized as synthetic polymer hydrogels or natural polymer hydrogels. Natural polymer hydrogels, characterized by their superior biocompatibility, responsiveness to environmental stimuli, abundant availability, and cost-effectiveness, have been identified as a promising and advantageous biomedical alternative. Polysaccharides, as essential components of natural polymers, have seen a surge in their application for the production of hydrogels in recent years. This has led to food-derived polysaccharide-based hydrogels (FPBHs) emerging as a promising prospect in the fields of food healthcare and medical pharmacy.

Polysaccharides are complex carbohydrates, composed of multiple monosaccharides connected by glycosidic bonds, forming polymeric high molecular weight carbohydrate chains. As a crucial component of all living organisms, polysaccharides maintain their homeostasis and play an indispensable role in the existence of them. Natural polysaccharides are a class of biological macromolecules, with glucan, chitosan, hyaluronic acid, alginate, and cellulose being the most commonly used polysaccharides in hydrogel synthesis. Polysaccharide materials, notable for their low cost, exhibit unique characteristics such as antibacterial functionality, facile chemical modification, superior biocompatibility, and biodegradability, making them a focal point of current research [5]. FPBHs confer numerous health benefits on humans, primarily because of the inherent biological activities of polysaccharides. Polysaccharides have been extensively documented to beneficially impact the gut microbiome by curtailing pathogens' growth, promoting the proliferation of probiotics, and enhancing host–microbial interactions [6]. The tunable hydrophilicity of hydrogels enables them to safeguard the bioactivity of polysaccharides within the gastrointestinal tract, facilitate the delivery and targeted release of medications in the circulatory system, and expedite skin surface repair.

Polysaccharide materials, such as sodium alginate, chitosan, hyaluronic acid, chondroitin sulfate, and carrageenan, currently play extensive and crucial roles in the field of biomedical research, particularly in wound healing, bone repair, cartilage regeneration, and arthritis management [7,8]. Furthermore, polysaccharides extracted directly from food-derived sources, including plants (e.g., *Oriental arborvitae*, Astragalus, and *Lonicera caerulea*), animals (e.g., Anthozoa, Sepiida, and Cetacean), and microorganisms (e.g., *Flammulina velutipes*, Tremella fuciformis Berk, and Flavobacterium sp.), can also serve as feedstocks for the synthesis of polysaccharide-based hydrogels. Certain animals, plants, or organisms inherently possess high nutritional value and serve as natural sources for the isolation and purification of specific polysaccharides. These polysaccharides exhibit distinctive biological activities that are exclusively found in a particular type of food. For instance, Tremella fuciformis Berk is the sole source of white tremella polysaccharides. Although similar fungi species may exist, such as *Auricularia auricula* (L.ex Hook.) Underwood, it is not possible to isolate polysaccharides with comparable structure and activity from these sources, thus highlighting the unique specificity of tremella polysaccharides. Numerous additional specific polysaccharides exist, renowned for their abundant and highly potent biological activities, which have been scarcely documented in previous retrospective studies.

Currently, no completed clinical trials evaluating the efficacy of polysaccharide-based hydrogels are available. The sole ongoing trial, investigating the use of alginate saline gel in Xijing Hospital of China, is currently recruiting participants through invitation (ClinicalTrials.gov identifier: NCT04781660). The safety and feasibility of an alginate saline gel-based repair method for heart failure were evaluated by implanting it into the left ventricle of patients, albeit without reporting the results. In contemporary clinical practice and research, hydrogels are primarily used as external application materials to serve as carriers for drug delivery, exemplified by their use in slow-release formulations for non-steroidal medications to treat herpes zoster, narcotics to alleviate labor pain, and antibiotics for the treatment of infected wounds. However, the investigation of FPBHs made from specific food-derived polysaccharides remains scant. To date, no clinical applications or retrospective analyses of fundamental experiments using these hydrogels have been reported.

To our knowledge, no systematic review has been published to date that overviews the biomedical applications of hydrogels made of natural food-derived polysaccharides. Polymerization of saccharides is an ongoing research area, with an increasing number of practical applications for newly discovered polysaccharide materials in tissue engineering. The food-derived polysaccharides discussed in this review pertain to the polysaccharides directly extracted from the natural food (a specific species, e.g., certain animals, plants, or microorganisms), which has specific biological activity and cannot be acquired from other categories of natural foods. The novelty of this study lies in its comprehensive critical appraisal of the existing literature on FPBHs, encompassing their classification, definition, formulation, characterization, and specific properties. The functional roles of FPBHs and their applications in biomedicine are expounded upon, laying the theoretical foundation for their translation into healthcare or clinical settings and charting a course for potential sustainable production.

## Search Strategy

**FIGURE 1:**
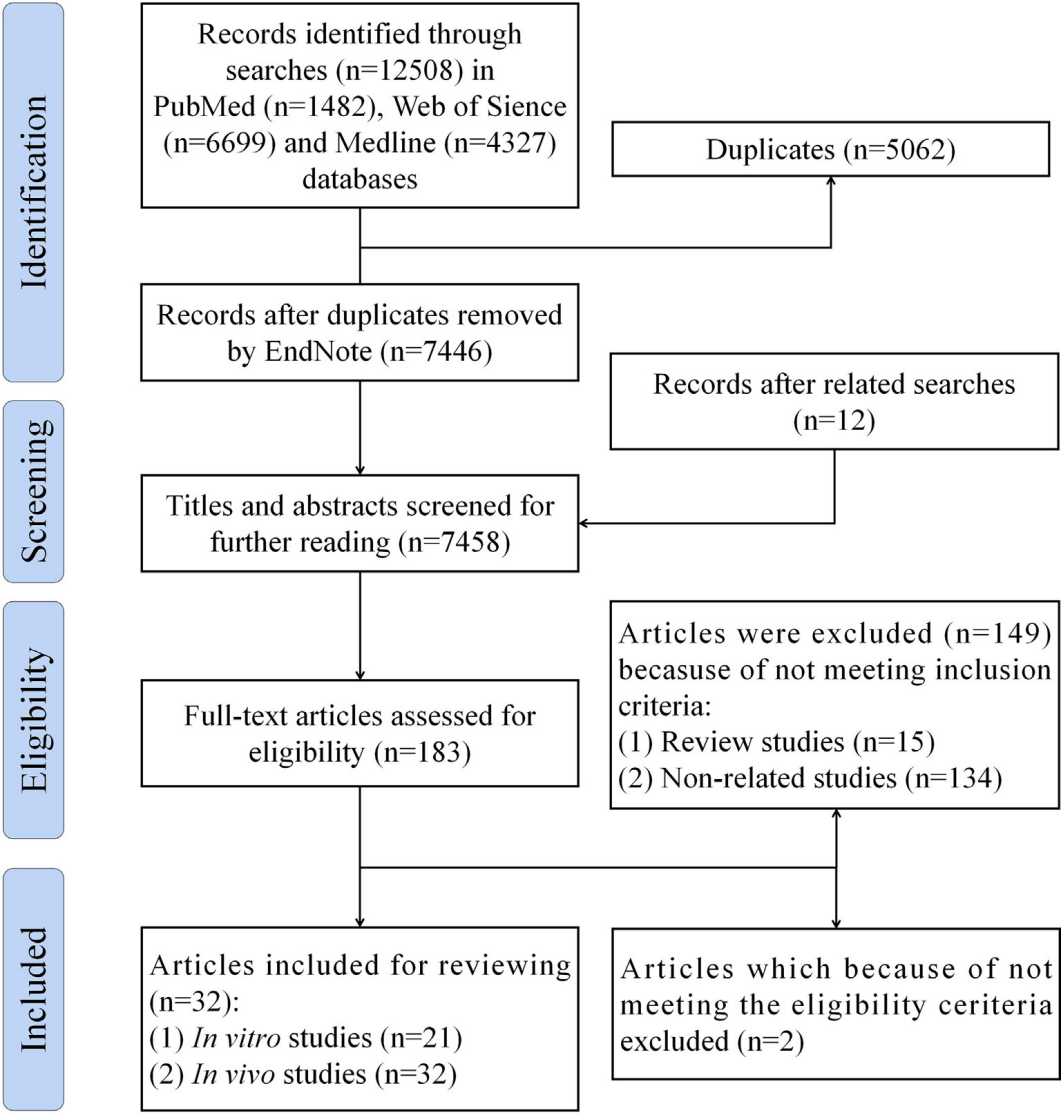
Flow diagram of the literature search and study selection process.

This systematic review examined the literature, retrieved from PubMed, Web of Science, and Medline databases, on FPBHs from inception to 23th Oct. 2024, utilizing the search terms “polysaccharide” and “hydrogels.” All studies related to the application of hydrogels in the biomedical field were included, and those that explored only the properties of hydrogels themselves were excluded. Furthermore, an exhaustive review of references and pertinent records was conducted. Two independent reviewers screened all included studies, and any discrepancies were resolved through consensus or by a third reviewer. The quality assessment of the included studies was performed by 2 reviewers, with any disagreements settled by consensus or a third reviewer's arbitration. The study selection process is schematically depicted in Figure 1. This systematic review synthesizes and analyzes the hydrogel raw materials, production methods, biomedical models, and biomedical effects of all the studies included.

## Selection of Studies

The electronic and manual searches yielded 6258 articles of potential relevance after removing duplicates and adding related research. Title and abstract screening were conducted to meet the inclusion criteria. A total of 183 studies were thoroughly analyzed to precisely assess for exclusion criteria, resulting in the exclusion of 149 articles. Ultimately, 32 articles [[9], [10], [11], [12], [13], [14], [15], [16], [17], [18], [19], [20], [21], [22], [23], [24], [25], [26], [27], [28], [29], [30], [31], [32], [33], [34], [35], [36], [37], [38], [39], [40]] were identified and included in this review. Among the 32 included articles, all conducted *in vitro* research, whereas 21 [9,[12], [13], [14],^16^,^17^,^19^,[21], [22], [23],[28], [29], [30], [31], [32], [33], [34], [35],[38], [39], [40]] performed *in vivo* studies.

### Sources and characteristics of food-derived polysaccharides

**FIGURE 2:**
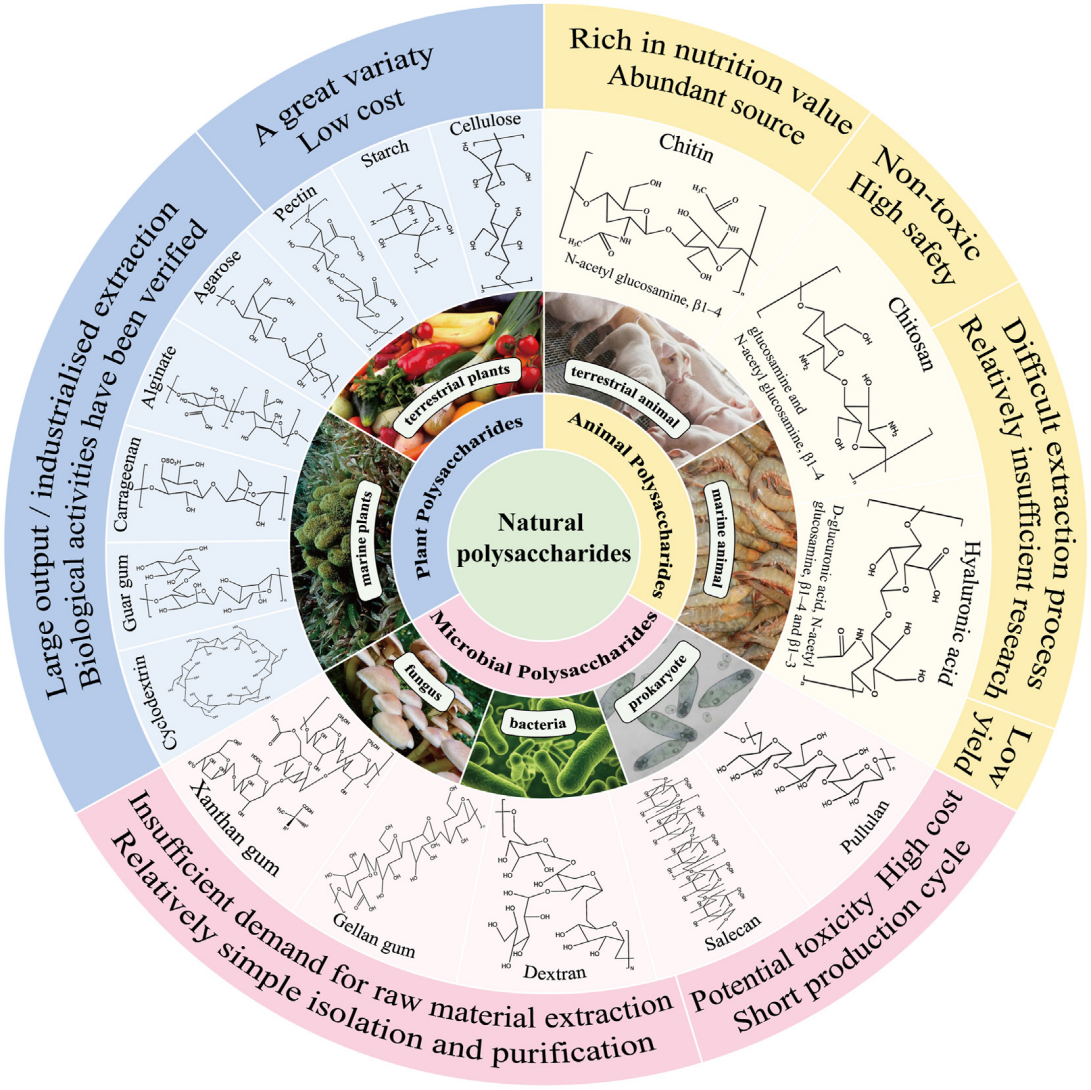
Classification, common types, and characteristics of natural polysaccharides.

Figure 2 presents the classification, common types, and characteristics of natural polysaccharides. The specificity of these polysaccharides’ physiological functions is intimately linked to their monosaccharide composition, molecular weight, configuration, and chain conformation. Polysaccharides derived from edible sources, known as food-derived polysaccharides, can be obtained exclusively from specific animal species, plants, or microorganisms and exhibit distinctive biological activities. These polysaccharides hold great potential for synthesizing hydrogels in the field of biomedical applications. These hydrogels, designated as FPBHs, exhibit significant versatility and have been extensively investigated for their potential applications in tissue engineering, drug delivery, and wound healing.

### Categorization of nature food-derived polysaccharide sources

Natural food-derived polysaccharides are ubiquitous in living organisms, including animals, plants, and microorganisms. Animal-derived polysaccharides are widely distributed and present in almost all animal tissues and organs, renowned for their abundant biological activity, complex structure, and potential for hydrogel formation. Polysaccharides of animal origin predominantly localize in the interstitial spaces of cells and encompass a broad range of compounds, such as glycogen, chitin, heparin, chondroitin sulfate, hyaluronic acid, keratin sulfate, acid mucopolysaccharides, and glycosaminoglycans. These polysaccharides, including chondroitin sulfate, hyaluronic acid, and keratin sulfate, are all glycosaminoglycans, a class of amino-containing polysaccharides that serve as the sugar chain component of proteoglycans.

Plant polysaccharides can be categorized as either terrestrial or marine. The cell walls of terrestrial plants, especially certain “Chinese herbs that can be added as food to the daily diet,” such as Lycium chinense Miller, Ziziphus jujuba Mill, Citrus reticulata Blanco peel, Cornu Cervi pantotrichum, etc., consist of various prebiotic polysaccharides, including cellulose, hemicelluloses, lignin, inulin, starch, pectin, and guar gum [8]. Polysaccharides found in most plants (except starch) are indigestible by the human body and serve as prebiotics to promote gastrointestinal health by curbing pathogens and stimulating the immune system.

Microbial sources of polysaccharides, which include bacteria, fungi, and prokaryotes, are classified into extracellular, cell wall, and intracellular varieties. Extracellular polysaccharides primarily consist of Gellan, xanthan, curdlan, pullulan, bacterial cellulose, welan gum, and hyaluronic acid. Cell wall polysaccharides primarily comprise lipopolysaccharides, lipopolysaccharides in bacteria, glucan, chitosaccharides, and their complexes in fungi. Intracellular polysaccharides are predominantly unique to microorganisms themselves, such as edible fungal polysaccharides (e.g., *Flammulina velatum*, Dictyophora, and Cordyceps Link) and bacterial (e.g., *Aspergillus flavus* and Lactobacillus) polysaccharides for fermentation.

### Source and characteristics of specific polysaccharides used in FPBHs

The polysaccharides selected for examination in the 32 FPBHs studies [[9], [10], [11], [12], [13], [14], [15], [16], [17], [18], [19], [20], [21], [22], [23], [24], [25], [26], [27], [28], [29], [30], [31], [32], [33], [34], [35], [36], [37], [38], [39], [40]] reviewed were exclusively derived from plant and microbial sources (Table 1). Plant polysaccharides exhibit diverse structures derived from various plant species in nature, resulting in a low cost and the potential for large-scale production. Polysaccharides from plants have a vast yet poorly understood structure-activity relationship, which impedes their precise utilization. Nevertheless, there is a vast expanse for exploration in plant polysaccharides, particularly based on the wealth of knowledge accumulated from Chinese herbal medicine. Additionally, microbial exopolysaccharides, due to their nontoxic, safe, short production cycle, and easy separation and purification characteristics, have increasingly become viable substitutes for plant and animal products. Nevertheless, the limited use of animal polysaccharides in hydrogel production could be attributed to the intricate structure of these polysaccharides and the considerable variations in extraction methods, which pose challenges for large-scale production, thereby restricting the application of animal polysaccharides in this domain.

**Table 1:** Structural features and properties of different food-derived polysaccharides.

polysaccharide	Source	molecular weight	Monosaccharide Composition	Biological activities	Reference
*Bletilla striata* polysaccharide (BSP)	Extracted from *Bletilla striata* in China	1.35×10^5^ Da	Glc: Man = 1.0: 2.4	Low toxicity, good biocompatibility, good biodegradability, non-immunogenicity, and ample availability	[9,12,17,19, 23,27,29,33,39,40]
*Tamarind Kernel* polysaccharide (TKP)	Extracted from *Tamarind Kernel* in India	1.15×10^5^-25×10^5^ Da	Gal: Xyl: Glc = 1:2.25:2.9	Biocompatibility, no toxicity, biodegradability	[10, 11,14]
Egyptian *Avena sativa L.* polysaccharides (ASP)	Extracted from *Avena sativa L.* grains in Egypt	NM	Ara: Rib: Xyl: Fru = 0.319: 0.266: 0.096: 0.053	Biocompatibility, antibacterial properties	[13]
Flammulina velutipes polysaccharide (FVP)	Extracted from *Flammulina velutipes* in China	NM	NM	Immune regulation, memory improvement and anti-tumor activity	[15]
Konjac glucomannan (KGM)	Extracted from Konjac in China	5×10^5^-200×10^5^ Da	Man: Glc = 1.4–1.6: 1	Biocompatibility, antibacterial property	[16]
Red marine microalga *Porphyridium sp.* polysaccharide (PSP)	Extracted from Porphyridium sp.	30×10^5^ -50×10^5^ Da	Xyl: Glc: Gal = 2.1:1.0:1.1	Biocompatibility, anti-inflammatory, antioxidant and antiviral functions	[18]
*Zingiber offcinale* polysaccharide (ZOP)	Extracted from Zingiber officinale	6.04×10^6^ Da (7.17%) 5.42×10^3^ Da (92.83%)	GlcA: GalA: Glc: Gal: Ara = 1.97: 1.15: 94.33: 1.48: 1.07	Oxidation resistance, anti-inflammatory, antibacterial property, anti-fatigue activity, fatigue resistance, good biocompatibility, and good biodegradability.	[20]
*Astragalus* polysaccharides (AP)	Extracted from *Astragalus* in China	20.58×10^5^ Da	Glc: Man: Ara: Xyl: GlcA: Rha = 12.83: 0.27: 0.71: 1.63: 1.04: 0.56	Low cytotoxicity, high biocompatibility, immune regulation and anti-tumor properties	[21]
Paramylon	Extracted from euglena	1.9 ×10^5^ Da	Glucan	Antioxidant, anti-inflammatory and angiogenic properties	[22]
*Ulvan* polysaccharide	Extracted frome *Ulva armoricana* in France	500 × 10^3^, 180 × 10^3^ Da	NM	Biocompatibility and moisture retention	[24]
*Strychnos potatorum L.* (SPL) seeds polysaccharide	Extracted from SPL seeds	1.28 × 10^4^ Da	GalN: Gal = 1: 1.7	Good biocompatibility, anticancer activity	[25]
Tragacanth gum (TG)	Extracted from Astragalus species in Pakistan	475.6 × 10^3^ Da	GalA: Gal: Rha: Ara: Glc: Man: Xly = 31.3:28.4:24.2:0.3:0.1:0.1:0.1	Good thermal stability, excellent solubility, and long shelf life	[26]
*M. haplocalyx Briq.* polysaccharide (MP)	Extracted from the aerial parts of *M. haplocalyx Briq.*	1.8 × 10^5^ Da	Ara: Glc = 0.9: 3.9	Antioxidant activity, inhibiting viral and bacterial inflammation	[28]
*L. japonica Thunb.* Polysaccharide (LP)	Extracted from the flower bud of *L. japonica Thunb.*	8.9 × 10^3^ Da	Ara: Man: Glc: Gal = 1.8: 1.0: 3.6: 3.7	Antibacterial, anti-inflammatory, antioxidant, hypolipidemic, anti-tumor and anti-diabetic properties, as well as immune regulation	[28]
*Aloe barbadenis* polysaccharide (ABP)	Extracted from *Aloe barbadenis* in China	3.40 ×10^5^ Da	Man: Glc: Gal = 97.4: 1.3: 1.3	Anti-inflammatory, antioxidant and moisturizing properties, good biocompatibility	[30]
Fucoidan (FU)	Extracted from Fucus vesiculosus, Undaria pinnatifida, Laminaria saccharina, or Cladosiphon okamuranus	3.26 ×10^5^ Da, 2.76 × 10^5^ Da	NM	Anticancer, antioxidant, immunoregulatory, antiviral, antithrombic, and anti-inflammatory properties	[31,32,[34], [35], [36], [37]]
Okra polysaccharide (OP)	Extracted from *Abelmoschus esculentus* pods in China	NM	NM	Immunomodulation, anti-inflammatory, antioxidant property	[38]

BSP, *Bletilla striata* polysaccharide; Glc, glucose; Man, mannose; TKP, *Tamarind Kernel* polysaccharide; Gal, galactose; Xyl, xylose; ASP, *Avena sativa L.* polysaccharides; NM, not mentioned; Ara, arabinose; Rib, ribose; Fru, fructose; FVP, *Flammulina velutipes* polysaccharide; KGM, Konjac glucomannan; PSP, *Porphyridium sp.* polysaccharide; ZOP, *Zingiber offcinale* polysaccharide; GlcA, glucuronic acid; GalA, galacturonic acid; AP, *Astragalus* polysaccharides; Rha, rhamnose; SPL, *Strychnos potatorum L.* ; GalN, N-acetylgalactosamine; TG, tragacanth gum; MP, *M. haplocalyx Briq.* polysaccharide; LP, *L. japonica Thunb.* polysaccharide; ABP, *Aloe barbadenis* polysaccharide; FU, fucoidan; OP, Okra polysaccharide.

The utilization of naturally occurring polysaccharides directly from food sources for hydrogel production has been comparatively underexplored, particularly with regard to animal- and microbial-derived polysaccharides. Polysaccharide materials present a challenge and complexity in overcoming their production limitations, unearthing their additional biological activities, and integrating these findings with hydrogel production research.

Thirty studies [[9], [10], [11], [12], [13], [14],[16], [17], [18], [19], [20], [21],[23], [24], [25], [26], [27], [28], [29], [30], [31], [32], [33], [34], [35], [36], [37], [38], [39], [40]] used plant polysaccharides, which were derived from *Zingiber officinale*, Astragalus species, Bletilla striata, the flower bud of *Lonicera japonica Thunb*, the aerial parts of *Mentha haplocalyx Briq*, Tamarind Kernel, *Aloe barbadenis*, *Strychnos potatorum* Linn (SPL) seeds, *Ulva armoricana*, *Porphyridium sp*., konjac, Egyptian Avena sativa L., Abelmoschus esculentus pods, and *Fucus vesiculosus* (or *Undaria pinnatifida*, *Laminaria saccharina*, *Cladosiphon okamuranus*). Two studies [15,22] used polysaccharides from microbial sources, the fungus *Flammulina velutipes,* and single-celled eukaryotes Euglena. Ten [9,12,17,19,23,27,29,33,39,40] of the studies used *Bletilla striata* to extract *Bletilla striata* polysaccharides (BSP) as one of the raw materials for making FBPHs. BSP shows low toxicity, good biocompatibility, good biodegradability, non-immunogenicity, and ample availability, playing a repairing role in skin, bones, joints, and other tissues. Six [31,32,[34], [35], [36], [37]] of the studies used fucoidan (FU) from *Fucus vesiculosus*, *Undaria pinnatifida*, *Laminaria saccharina*, or *Cladosiphon okamuranus* to manufacture FBPHs. FU performs anticancer, antioxidant, immunoregulatory, antiviral, antithrombic, and anti-inflammatory properties. Three studies [10,11,14] have used Tamarind Kernel polysaccharides (TKP) to produce FBPHs that can play a beneficial role in wound healing and promote osteogenic differentiation. The demonstration of good biocompatibility and low toxicity enables TKP to exhibit anti-inflammatory, antioxidant, anti-tumor, and immunomodulatory functions to varying degrees.

The inherent properties of polysaccharides derived from diverse sources dictate their distinct biological activities, thereby facilitating the development of hydrogels fabricated from these polysaccharides for therapeutic applications in various clinical settings. This warrants attention and represents a promising avenue for exploration in the tissue engineering field.

### Preparation methods of polysaccharide-based hydrogels

**FIGURE 3:**
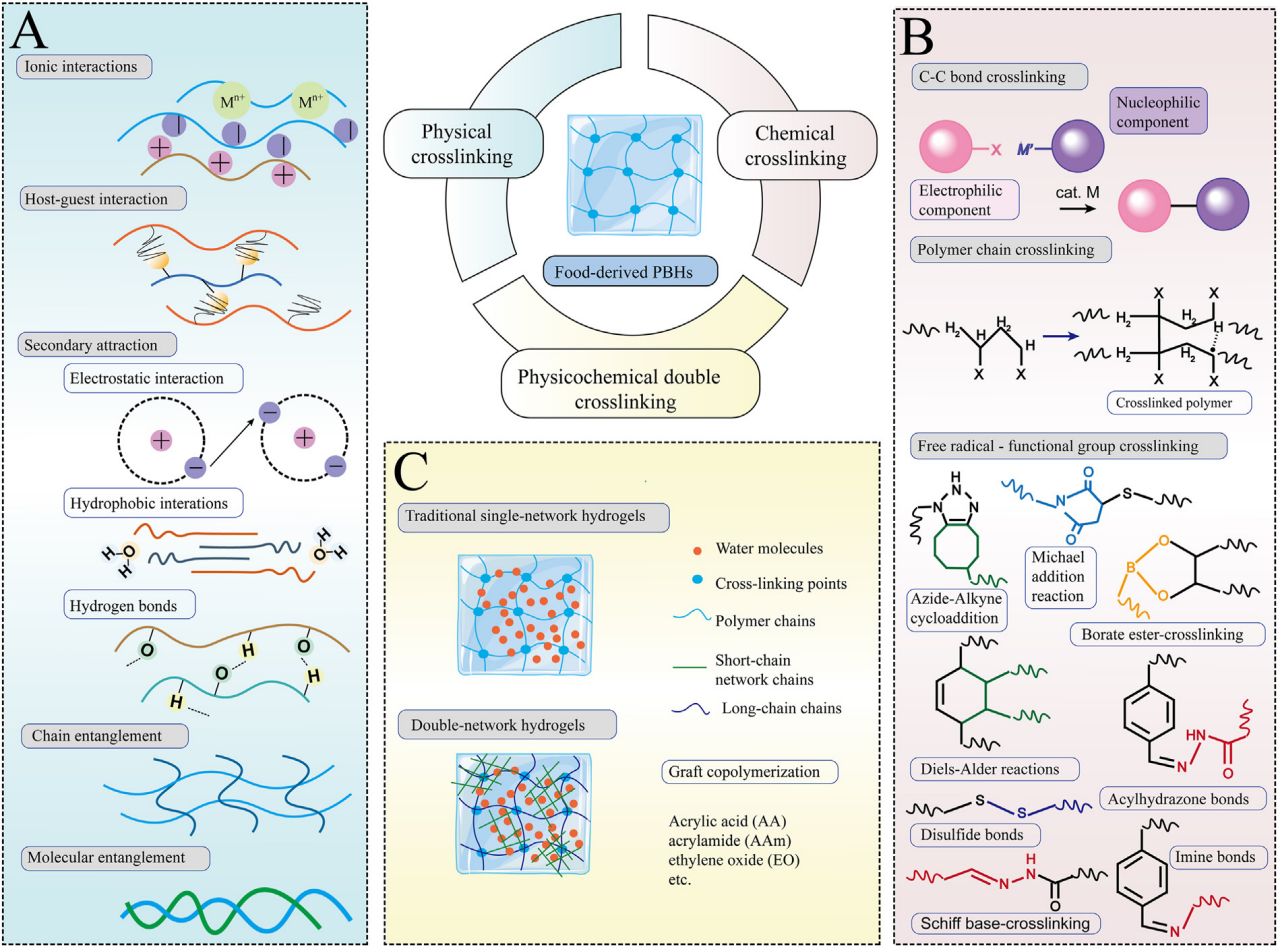
Synthesis of food-derived polysaccharide-based hydrogels polysaccharides. (A) Types and methods of physical crosslinking. (B) Types and methods of chemical crosslinking. (C) Methods for the formation of physicochemical double-crosslinked hydrogel networks.

The fabrication of FPBHs particles involves the combination of bioactive ingredients and polymeric excipients, yielding spherical droplets or particles. The size and size distribution of the resulting microparticles are the 2 primary variables that dictate the release of the bioactive compounds [41], which are influenced by the method of hydrogel preparation. Hydrogels are categorized based on their crosslinking mechanisms and properties into physical and chemical crosslinking methods. Consequently, FPBHs can be further subdivided into physical crosslinked hydrogels, chemical crosslinked hydrogels, and physically-chemically hybrid double-crosslinked hydrogels [42]. The production methods of FPBHs are shown in Figure 3. The hydrogel preparation methods and other materials except food-derived polysaccharides of the 32 studies included in this review are shown in Table 2.

**Table 2:** Hydrogel preparation method and biological effects *in vitro* of FPBHs.

Effect	Polysaccharide,	Other ingredients	Hydrogel preparation Method	Model	Biological responses	Reference
Biocompatibility Effect of promoting osteogenesis	BSP	Acetic anhydride, pyridine	BSP was acetylated to acBSP using pyridine and acetic anhydride. Different forms of acBSP material could be prepared through anti-phase transition method.	Human monocyte THP-1 cells line and mouse L929 fibroblasts line, human MSCs	The acBSP hydrogel efficiently facilitated the adhesion and activation of macrophages and notably induced the macrophages to express pro-osteogenic / -angiogenic genes.	[9]
Biocompatibility Effect of promoting osteogenesis	TKP	AA, free radical initiator Benzoyl peroxide	Briefly TKP was dissolved in distilled water to make 3% (w/v) solution. Different mole ratio of AA with respect to TKP was added to the TKP solution along with PP as radical initiator for the TKP-AA hydrogel.	Mouse and rat BMMs; RCOs; mouse MSCs; RAW264.7 cells line; HUVECs; Saos-2 cells line; F-11 cells line; mouse preosteoblast cell line MC3T3-E1	The TKP-AA hydrogel was biocompatible with HUVEC, F-11, Saos-2, Raw 264.7, RCOs, BMMs and MSCs. In the absence of any osteogenic inducing factors, the hydrogel surface enhanced the expression of different osteogenic genes in Saos-2 cells and MC3T3-E1 cells; additionally, it exhibited a pronounced effect on enhancing the differentiation of MSC-derived primary osteoblasts, although concurrently promoting cell adhesion, growth, and maturation.	[10]
Effect of promoting osteogenesisBiocompatibility	TKP	AA, free radical initiator Benzoyl peroxide	Briefly TKP was dissolved in distilled water to make 3% (w/v) solution. Different mole ratio of AA with respect to TKP was added to the TKP solution along with PP as radical initiator for the TKP-AA hydrogel.	F-11, Saos-2, RAW 264.7 cells line; mouse and rat BMMs; mouse MSCs	The TKP-AA hydrogel promoted the proliferation of F-11, Saos-2, RAW 264.7 cells, BMMs, and MSCs; additionally, it enhances osteogenic differentiation in MSCs.	[11]
Biocompatibility	BSP	CMC, CBM_940_	CMC and CBM_940_ were mixed in different concentration ratios, the PH was adjusted to 7 by triethanolamine, and then added into BSP solution to prepare BSP/CMC/CBM_940_ hydrogel.	Mouse fresh red blood cells, M293T cells line	The BSP/CMC/CBM_940_ hydrogel promoted the normal growth and proliferation of M293T cells, and effectively removes hydroxyl free radicals from blood cells without hemolysis.	[12]
Antibiosis	Egyptian ASP	PVA, Na CMC, HPMC, HEC, CBM_940_ and PVP	The PVA-ASP hydrogel was prepared by adding AP to aqueous PVA solution and then adding the mixed solution of polymer and bacitracin zinc, followed by repeated freeze-thaw cycles after ultrasonic treatment.	gram-positive (*S. aureus* and *M. leutus*) and gram-negative (*E. coli* and *P. aeruginosa*)	The PVA-ASP hydrogel showed antibacterial activity against *S. aureus* and *M. leutus* and was able to organize microbial penetration into the hydrogel.	[13]
Biocompatibility	TKP	AA, free radical initiator Benzoyl peroxide	Briefly TKP was dissolved in distilled water to make 3% (w/v) solution. Different mole ratio of AA with respect to TKP was added to the TKP solution along with PP as radical initiator for the TKP-AA hydrogel.	Mouse NIH/3T3 cells line, human keratinocyte HaCat cells and F-11 cells line	The TKP-AA hydrogel not only promoted the proliferation of co-cultured HaCat and F11 cells, but also promoted the formation of cell-cell contact.	[14]
Anti-inflammatory effectBiocompatibility	FVP	SPI, Glucono-delta-lactone powder	The FVP solution prepared at room temperature and SPI solution prepared at high temperature were mixed and heated, and finally the Glucono-δ-lactone powder was added to form the SPI-FVP gel.	Mouse RAW264.7 cells line	The SPI-FVP hydrogel promoted the proliferation and phagocytosis of macrophages with increased expression of IL-6, IL-10 and TNF-α.	[15]
Antibiosis Biocompatibility	KGM	γ-PGA, EDC, ADH	NH_2_ solution was prepared from ADH-substituted γ-PGA by an amide condensation reaction. Oxidation of KGM was achieved using NaIO_4_. P-NH_2_ and oxidized KGM were dissolved in different concentrations of deionized water, and the 2 solutions were rapidly mixed by a vortex oscillator at a constant final volume to form a hydrogel (P-OK).	RAW 264.7 cells line and NIH/3T3 cells line; *S. aureus* and *E. coli*	NIH-3T3 cells and RAW264.7 cells could proliferate normally on the P-OK hydrogel. P-OK hydrogel can stimulate the secretion of IL-10 in RAW264.7 cells; it inhibited the growth of bacteria and has good antibacterial effect.	[16]
Biocompatibility	BSP	PEG, PCL, AAM	Dried PCL, PEG and isophorone diisocyanate were reacted with chain extender and t9 and t12 catalysts to obtain WPU emulsion. The hydrogel was obtained by mixing wpu emulsion, AAM, poly(ethyleneglycol) dimethacrylate, potassium persulfate and different amounts of BSP.	Rabbit whole blood, NIH/3T3 cells line	The hydrogel reduced the probability of hemolysis and was less cytotoxic to NIH3T3.	[17]
Effect of promoting osteogenesisBiocompatibility	PSP	FmocFF peptide	The FmocFF peptide was dissolved in DMSO solvent. FmocFF peptide solution was vortex mixed with PSP solution in different ratios to obtain FmocFF/PSP composite hydrogels.	MC3T3-E1 osteoblasts line	MC3T3-E1 osteoblasts could grow and proliferate on the FmocFF/PSP hydrogel and increased calcium deposition, indicating osteogenic ability.	[18]
Biocompatibility Antibiosis	BSP	Methylcellulose and methylparaben	The hydrogel was synthesized by combining BSP and methylcellulose with 0.04% methyl methylparaben in a self-assembly reaction.	Fresh white rabbit red blood cells, L929 cells line; Bacteria *S. aureus* and *E. coli*	The hydrogel has antibacterial activity and no obvious hemolytic effect on fresh white rabbit red blood cells; in addition, L929 cells were able to grow and proliferate on it.	[19]
Biocompatibility	ZOP	CS, epichlorohydrin	CS and ZOP were mixed in urea aqueous solution and then mixed with epichlorohydrin as a crosslinker.	Mouse RAW264.7 cells line	The hydrogel showed no cytotoxicity	[20]
Antitumous effect	AP	HA, Apatinib	The Cu - Apatinib copper complex was loaded into oligomeric HA-polymeric micelles and subsequently embedded into an AP hydrogel.	Mouse melanoma cell B16-F10 cells line	The hydrogel inhibited the growth and proliferation of melanoma cells.	[21]
Anti-inflammatory effect	Paramylon, 98%	1,4-butanediol diepoxyglycerol ether	Paramylon powder and sodium hydroxide were added to water and stirred thoroughly. Then 1,4-butanediol diepoxyglycerol ether was mixed completely into the solution, and dialyzed for times.	RAW264.7 cells line induced by 10 ng/mL LPS	The hydrogel could inhibit LPS-induced macrophage inflammation and reduce the levels of inflammatory factors TNF-α and IL-7.	[22]
Anti-inflammatory effectBiocompatibility	BSP	MA	MA was added into the gelatin solution and BSP solution to make GelMA and BSPMA solution respectively. Then the GelMA and BSPMA solution were mixed and irradiated with ultraviolet rays after adding a photoinitiator to make BSPMA/GelMA Dual-Cross-Linked (B–G) Hydrogels.	NIH/3T3 cells line, RAW264.7 cells line	The B-G hydrogel could effectively regulate the M1/M2 phenotype of macrophages, significantly promote the proliferation and migration of fibroblasts.	[23]
Antibiosis	*Ulvan* polysaccharide	MCC, CNCs	CNCs were isolated from MCC by sulfuric acid hydrolysis. AgNO_3_ aqueous solution was added with CNCs or *ulva* polysaccharide, then aqueous solution of NaBH_4_ was added, and the AgNPs hydrogel was obtained by dialysis drying.	*E. coli*, *P. aeruginosa* and *S. aureus*	AgNPs colloids showed good stability in PBS and strong antimicrobial activity, especially showing stronger resistance to Gram strains.	[24]
Antitumous effect Antibiosis	SPL seeds polysaccharide	MBA, *C. spinarum Aqueous* Leaf Extract, Encapsulation of 5-FU	APS, SPL and MBA were mixed to make SPL-DMA Semi-IPN Hydrogels. Then AgNO and *C. spinarum* Aqueous Leaf Extract were added to them to form Green Synthesis of SPL-DMA-Ag Nanocomposite Hydrogels.	Human HeLa and NIH/3T3 cells lines, bacteria, i.e., E. coli, P. aeruginosa, S. aureus, and K. pneumonia	5-FU-loaded hydrogels showed inhibiting the growth and proliferation of HeLa and NIH/3T3 cells; the hydrogels without 5-FU shows antibacterial characters.	[25]
Biocompatibility	TG	CLPs, TOCNF	CLPs dispersion, TOCNF, and TG were mixed with different ratio to prepare hydrogel inks, which were used by a bioprinter.	HepG2 cells line	The hydrogel showed no cytotoxicity and the ability to promote cell proferation.	[26]
Biocompatibility	BSP	APS, MBA	APS was added to BSP solution and kept for 5 minutes to produce BSP macroradical, MBA was put into a reaction system in a nitrogen atmosphere to get BSP-g-PAA solution. The pre-dissolved PVA solution was added to the BSP-g-PAA solution.	Rabbit whole blood; L929 fibroblasts	*In vitro* blood compatibility test showed that the hydrogel had low hemolysis rate and good coagulation effect. The material was not cytotoxic to L929 cells.	[27]
Biocompatibility	LP and MP	CS	A mixture of MA and isopropanol was added dropily to a basic solution of CS, desalted, filtered and dialyzed to obtain O-CCS. The mixture of LP and MP (3:1) was dissolved in 50% ethanol and redissolved in a solution of nitrogen oxide. Ethylene glycol, NaIO_4_ and NaCl were added successively to obtain OLMP. Finally, OPHs were prepared by mixing O-CCS and OLMP.	Mouse progastric cancer MFC cells line	MFCs could proliferate normally and spread well on the surface of the OPHs, and the hydrogel had no toxic effect on cells.	[28]
Biocompatibility	BSP	CFs	CFs and BSP were dissolved in acetic acid and stirred to obtain CFOB, and then F-107 and PVP were dissolved in them to obtain COF hydrogel.	Human fresh blood cells, fibroblasts L929 cells line	The COF hydrogel showed the ability to promote fibroblasts proliferation, and not to lead to increased hemolysis.	[29]
AntibiosisBiocompatibility	*Aloe barbadenis* polysaccharide (ABP)	PMP, TFA, D_2_O, PVA	ABP and honey were added to the PVA solution, heated and stirred for 30 min and then borax solution was added. The final solution was molded and bubbles removed in a Petri dish, followed by 2 cycles of freeze-thaw to obtain the ABP/Honey@PVA hydrogel.	NIH/3T3 cells line and L929 fibroblasts line; *E. coli*, *S. aureus* and fungus *C. albicans*	The ABP/Honey@PVA hydrogel showed excellent biocompatibility with NIH-3T3 cells and L929 cells, and showed significant growth inhibition against *S. aureus*, *E. coli*, and *C. albicans*.	[30]
BiocompatibilityAntitumous effectAntibiosis	FU	CMC, TA, HAuCl_4_·4H_2_O	The TA and HAuCl_4_ solution were mixed to obtaine TA-modified gold nanoparticles (AuNPs@TA PMN). The FU and the sodium periodate was mixed. The resulting mixture underwent dialysis in deionized water for 3 days before being freeze-dried to obtain the oxidized FU. Multiple concentrations of AuNPs@TA PMN were dispersed in deionized water and added to the oxidized FU solution. Finally, CMC was incorporated to form a CMC/OF/AuNPs@TA hydrogel.	L929 fibroblasts, B16-F10 cells line, fresh mouse whole blood cells; *E. coli*, *S. aureus*	The CMC/OF/AuNPs@TA hydrogel demonstrated a viability of over 70% for L929 fibroblasts, exhibited <5% hemolysis in blood cells, and effectively suppressed the proliferation of B16 cells. Furthermore, it displayed potent antibacterial activity against *E. coli* and *S. aureus*.	[31]
BiocompatibilityAnti-inflammatory effect	FU	Dextran, MA,	The DexMA was synthesized by combining dextran with glycidyl methacrylate ethyl methacrylate, and a solution was prepared by adding DexMA to the photoinitiator phenyl-2,4,6-trimethylbenzoylphosphonate lithium. FU was incorporated into the solution via UV irradiation to fabricate the FU-DexMA composite hydrogel.	Nasopharyngeal carcinoma NPC cells	The FU-DexMA hydrogel demonstrated the ability to enhance cell viability of NPC cells, attenuate intracellular inflammatory response.	[32]
Biocompatibility	BSP	Gelatin	Dialdehyde BSP (BSP-CHO) was synthesized by Malaprade reaction with periodic salt, gelatin was modified with ethylenediamine, and the 2 were mixed to form BG-gel.	L929 fibroblasts; Rat whole blood cells	The hydrogel was not toxic to L929 cells, and the hemolysis rate was <5%.	[33]
Effect of promoting osteogenesisBiocompatibility	FU	Nap-FFGRGD peptide	The Nap-FFGRGD peptide and the Na_2_CO_3_ solution was mixed, and then was added to the FU-containing solution. The pH of the solution was adjusted to 7.4, followed by a heating and cooling process that resulted in the formation of a self-assembling glycopeptide hydrogel.	Primary rabbit articular chondrocytes	The Nap-FFGRGD /FU hydrogel demonstrates the ability to enhance primary rabbit articular chondrocyte viability and promote cytoskeletal augmentation, thereby exhibiting a favorable osteogenic effect.	[34]
BiocompatibilityAntibiosis	FU	Alginate, SDA, CA	The CA/FU mixture is prepared by combining sodium alginate with FU and CA. *Lactobacillus rhamnosus* is incorporated into the mixed hydrogel precursor (CA/FU mixture). The mixed hydrogel precursors are immersed in a 2.5% (m/v) solution of D-(+)-gluconate δ-lactone to form the hydrogel.	L929 fibroblasts; *S. aureus* and *C. albicans*	The hydrogel had no obvious toxicity to L929 cells, and could inhibit the growth of *S. aureus* and *C. albicans*.	[35]
BiocompatibilitySecretory capacity	FU	PVA, MA, ICEMA	The PVA modified with methacrylate was obtained by incorporating ICEMA into a PVA-DMSO solution. The FU was dissolved in milliq water, followed by the addition of DMSO and excess MA. A photoinitiator at a concentration of 0.05 wt% was introduced to the PVA-MA and fucoidan-MA solution, and under ultraviolet light to obtaine the PVA-fucoidan-MA hydrogel.	Mouse insulinoma cells (MIN6)	The PVA-fucoidan-MA hydrogel not only increased the cell viability of MIN6 cells and reduced apoptosis, but also promoted the cells to secrete more insulin.	[36]
Effect of promoting osteogenesisBiocompatibility	FU	PRF, CS	The CS was dissolved in hydrochloric acid, followed by the addition of FU/aqueous and CS solution. The sample was then subjected to another round of lyophilization, after which pure platelet-rich fibrin was added to yield PRF/FU_CS hydrogel.	Fresh human whole blood cells, human MSCs derived from bone marrow	The PRF/FU_CS hydrogel promotes the release of growth factors (TGF-β, VEGF, IL-8 and EGF) in whole blood cells and enhance the cell viability of hMSCs.	[37]
Biocompatibility	OP	XG	The XG was dissolved in distilled water to obtain a 4% (w/v) solution, which was then combined with the OP solution of 2%, 4%, and 6% (w/v) prepared from distilled water. Subsequently, a 1% (w/v) borax solution was added and thoroughly stirred to achieve uniformity, resulting in the formation of OP/XG hydrogel.	L929 fibroblasts and HUVEC cells line; fresh rat whole blood cells	The XG/OP hydrogel demonstrated the ability to enhance cell viability in L929 and HUVEC cells. The hemolysis rate of whole blood cells remains below 5%. Additionally, it promoted the upregulation of skeletal proteins in HUVEC cells.	[38]
BiocompatibilityAntibiosis	BSP	gelatin, ADH	The oxidized BSP was synthesized using the oxidizing agent NaIO_4_ to form OBGTP. ADH was subsequently incorporated into gelatin to create ADH/Gel, and finally, the 2 components were combined to produce OBGTP/ADH hydrogel.	Fresh SD rat whole blood cells, L929 fibroblasts; *E. coli* and *S.aureus.*	The hemolysis rate was found to be below 5%, although the cell survival rate exceeded 80%. Furthermore, the hydrogel exhibited inhibitory effects against *E. coli* and *S.aureus*.	[39]
Biocompatibility	BSP	HA, Zein, CS, β-GP, Puerarin	The BSP solution was mixed with NaIO_4_ for dialysis to obtain oxidized BSP. Acetic acid was added to the prepared suspension of HA-SH-zein nanoparticles. The OBSP/CS hydrogel was obtained by dissolving an appropriate amount of CS and oxidized BSP in the suspension, followed by the addition of β-GP solution.	L929 fibroblasts; fresh pig colon	The OBSP/CS hydrogel could enhance the cell viability of L929 cells without showing toxicity, and has obvious retention effect on pig colon *in vitro* and good adhesion.	[40]

AA, acrylic acid; AAM, acrylamid; ABP, *Aloe barbadenis* polysaccharide; ADH, adipic acid dihydrazide; AP, *Astragalus* polysaccharides; APS, Ammonium persulfate polyacrylic; ASP, *Avena sativa L.* polysaccharides; β-GP, β-sodium glycerophosphat*e;* BMMs, bone-marrow derived preosteoclasts; BSP, *Bletilla striata* polysaccharide; CA, calcium carbonate; *C.albicans*, *Candida albicans;* CBM_940_, Carbomer 940; CFs, collagen fibers; CLPs, colloidal lignin particles; CMC, carboxymethyl chitosan; CNCs, cellulose nanocrystals; CS, chitosan; *E. coli, Escherichia coli*; FmocFF, Lyophilized Fmoc-Phe-Phe-OH; FU, fucoidan; FVP, *Flammulina velutipes* polysaccharide; HPMC, hydroxypropyl methylcellulose; KGM, Konjac glucomannan; SPL, *Strychnos potatorumL.*; TG, tragacanth gum; MP, *M. leutus*, *Micrococcus leutus; M. haplocalyx Briq.* polysaccharide; MSCs, mesenchymal stem cells; K. pneumonia, Klebsiella pneumonia; LP, *L. japonica Thunb.* polysaccharide; OP, Okra polysaccharide; P. aeruginosa, Pseudomonas aeruginosa; PMP, 1-phenyl-3-methyl-5-pyrazolone; PSP, *Porphyridium sp.* polysaccharide; HEC, hydroxyethylcellulose; HUVECs, human umbilical vein endothelial cells; PRF, pure platelet-rich fibrin; PVP, polyvinylpyrrolidone; PEG, polyethylene glycol; SPI, soy protein isolate; γ-PGA, γ-polyglutamic acid; EDC, 1-(3-dimethylaminopropyl)-3-ethylcarbodiimide; PCL, polycaprolactone; HA, hyaluronic acid; MA, type A gelatin, methacrylic anhydride; MCC, microcrystalline cellulose; MBA, N,N-methylene bisacrylamide; 5-FU, 5-Fluorouracil; TFA, trifluoroacetic acids; TKP, *Tamarind Kernel* polysaccharide; TOCNF, TEMPO-oxidized cellulose nanofibril; SDA, sabouraud dextrose agar; ICEMA, 2-isocyanatoethyl methacrylate; XG,xyloglucan; RCOs, rat calvarial osteoblasts; *S.aureus*, *Staphylococcus aureus*; TA, tannic acid; ZOP, *Zingiber offcinale* polysaccharide.

## Physical Crosslinking

Twelve studies [12,13,15,18,19,21,29,30,34,35,37,38] employed exclusive physical crosslinking techniques to synthesize FPBHs. Physical crosslinking alters the physical entanglement of polymer chains or establishes a hydrogel network via noncovalent interactions (Figure 3A). The hydrogel network is sustained by molecular entanglement, chain entanglement, and secondary attractions (including electrostatic interaction, hydrogen bonding, or hydrophobic interactions), without the formation of any covalent bonds throughout this process. The gentle nature of natural polysaccharides allows for the preparation of FPBHs through physical crosslinking under straightforward conditions.

**FIGURE 4:**
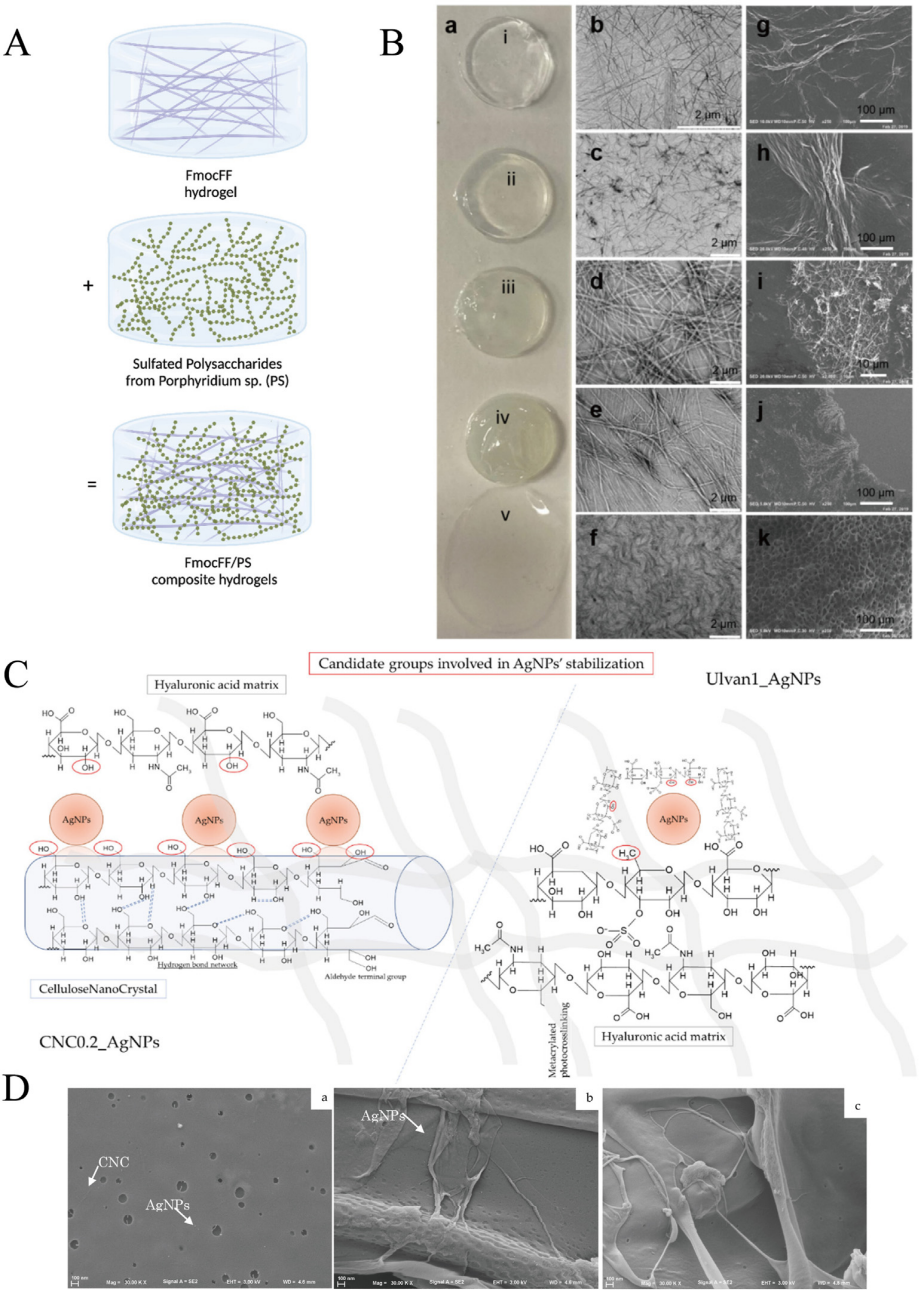
Schematic illustration and images of 2 food-derived polysaccharide-based hydrogel examples. (A) FmocFF peptide and PSP formation into FmocFF/PSP hydrogels [18]. B. Appearances and electron microscope images of the FmocFF/PSP hydrogels. (a) Appearance image of (i) FmocFF peptide, (ii) FmocFF/PSP 5 mg/mL, (iii) FmocFF/ PSP 10 mg/mL, (iv) FmocFF/ PSP 20 mg/mL, (v) pure PSP. (b-f) SEM images of (b) FmocFF, (c) FmocFF/ PSP 5 mg/mL, (d) FmocFF/ PSP 10 mg/mL, (e) FmocFF/ PSP 20 mg/mL, (f) pure PSP. (g–j) TEM images of (g) FmocFF, (h) FmocFF/ PSP 5 mg/mL, (i) FmocFF/PSP 10 mg/mL, and (j) FmocFF/PSP 20 mg/mL. (k) SEM of the lyophilized pure PSP [18]. (C) Schematic representation of the developed hydrogel systems. Candidate groups involved in AgNP interactions have been underlined in red [24]. (D) Scanning electron microscopy images of different hydrogels incorporating [24]. (a) Met-HA-CNC0.2_AgNPs; (b) Met-HA-Ulvan1_AgNPs; (c) Met-HA-Unload. (A, B: Reproduced with permission from reference #18, Copyright of ©2022 MDPI; C, D: Reproduced with permission from reference #24, Copyright of ©2022 MDPI.)

The study by Halperin-Sternfeld et al. [18] primarily involves the use of *Porphyridium sp.* polysaccharides (PSP) from a red marine microalga. The hydrogel was fabricated by combining aqueous solutions of PSP and dimethyl sulfoxide (DMSO) with FmocFF peptide solutions in varying ratios at room temperature. As illustrated in Figure 4A, this method represents a typical physical crosslinking approach for the preparation of hydrogels, capable of generating self-supporting and homogeneous gel structures. As revealed by transmission electron microscopy and scanning electron microscopy analyses (Figure 4B), the hydrogel exhibits a nanofiber-like morphology, with different ratios of its components leading to distinct structural states. Taking the FmocFF/PSP hydrogel as an example, although physically crosslinked hydrogels exhibit low cytotoxicity and are suitable for encapsulating delicate biological activity or living cells, they also present some limitations such as poor mechanical properties, lack of long-term stability, and susceptibility to degradation.

## Chemical Crosslinking

Among the 6 articles [10,11,14,20,22,24] exclusively employing chemical crosslinking methodology for the production of FPBHs, it is noteworthy that 3 studies [10,11,14] have utilized TKP-acrylic acid (AA) hydrogel as a research material. Chemical crosslinking hydrogels are formed by introducing crosslinking agents to facilitate the formation of new covalent bonds between polymer molecular chains (Figure 3B). A crosslinking agent refers to a compound possessing more than 2 reactive functional groups that can effectively cross-link polymers and establish intermolecular bonds. Other commonly employed crosslinking agents include compounds containing difunctional or multifunctional groups, such as glutaraldehyde (GLA), EPichlorohydrin (EPI), N, N'-methylene diacrylamide (MBA), ethyldimethacrylate (EGDMA), polyvalent metal ions, etc. There are 3 primary approaches for the formation of chemical crosslinking hydrogels: (1) C-C bond crosslinking in which free radicals generated by crosslinking agents initiate chain reactions among polymers; (2) polymer chain crosslinking, involving the reaction between functional groups of the crosslinking agent and the polymer to establish molecular connectivity within the polymer chains; (3) free radical–functional group crosslinking, where a combination of free radical initiation and reaction between the functional groups of the crosslinking agent leads to covalent bonding [43].

Additionally, photoactivation serves as a chemical crosslinking method enabling the formation of hydrogels through co-existing photoinitiators such as methacrylate or arylazide modified macromolecular monomers [44]. Enzyme-mediated cross-linking is also employed as a chemical approach using horseradish peroxidase, a single-chain β-heme protein capable of catalyzing phenol or aniline derivative coupling through hydroperoxide decomposition to facilitate cross-linkage [45]. Furthermore, exploration into utilizing natural polysaccharides as potential crosslinking agents for hydrogel synthesis remains unexplored in existing studies. Jing et al. [20] used epichlorohydrin as a crosslinking agent to make CS and ZOP into hydrogels, which is a chemical crosslinking method by which hydrogen atoms in the alkyl chain are replaced by hydrogen ions to form hydrogen and oxygen ions. The mechanical properties of chemical crosslinked hydrogels are commendable; however, their apparent cytotoxicity poses a major challenge to practical application and may leave the nutritional value of the polysaccharide components in hydrogels unutilized.

## Physicochemical Double Cross-linking

In fourteen studies [9,16,17,23,[25], [26], [27], [28],[31], [32], [33],^36^,^39^,^40^], physicochemical double cross-linking methods were used to produce FPBHs. Usually, one or more hydrogels were prepared separately by chemical crosslinking method, and then the hydrogels were physically mixed with other hydrogels or other materials. Both physical and chemical cross-linking exist in the three-dimensional network of physical-chemical hydrogels, and 2 kinds of interpenetrating polymer networks are formed by ion-covalent double cross-linking mechanism. Graft co-polymer method: by changing the type of monomer, branch chain molecular weight, grafting rate can effectively improve the adsorption capacity of hydrogel and make it have selectivity. Commonly used graft monomers are groups containing carbon-carbon double bonds and active adsorption functions, including acrylic acid (AA), acrylamide (AAm), ethylene oxide (EO), etc.

Polez et al. [26] first obtained colloidal lignin particles dispersion, TEMPO-oxidized cellulose nanofibril, and Tragacanth gum by chemical crosslinking method, and then mixed them by physical crosslinking method to make a double-crosslinked hydrogel. Massironi et al. [24] employed a more intricate chemical crosslinking strategy, wherein they opted for a blend of ulvan or cellulose nanocrystals as the stabilizer for the reaction, and utilized sodium borohydride to reduce AgNO into silver nanoparticle AgNPs hydrogels, shown in Figure 4C,D. Compared to single crosslinked hydrogels, double-crosslinked hydrogels exhibit enhanced mechanical strength and toughness, although mitigating potential cytotoxicity concerns. However, the preparation process of double-crosslinked hydrogels is intricate, incurs high costs, and exhibits poor repeatability.

### Attributes and characterization of FPBHs

The application efficacy of FPBHs in biomedicine is determined by their attributes and characteristics, which are commonly evaluated based on physical properties (such as form, structure, and shape), chemical properties (including composition, content, and chemical structure), and functional properties (such as rheology, water absorption, and stability).

### Physical properties: form, structure, and shape

FPBHs have various physical properties, among which form, structure, and shape are the most important. The FPBHs is a gel-like substance in terms of its form, capable of exhibiting rheological properties under the influence of external forces (like gravity, pressure, etc.) and transitioning into a soft gel state. This gel state can be regulated by adjusting the concentration of polysaccharide molecules, temperature, pH value, and other factors to achieve the needs of different application scenarios. FmocFF/PSP hydrogel made by Halperin-Sternfeld et al. [18], as depicted in Figure 4B (i-v), exhibited distinct gel states with varying textures and proportions.

In addition, FPBHs possess both viscosity and plasticity, enabling them to adapt to the application requirements of diverse morphologies and structures. Regarding structure, the architecture and morphology of FPBHs can be regulated through various preparation methods and conditions. This allows for the formation of different structural forms such as nanoparticles [24], microspheres [46], fibers [47], and films [48]. These distinct forms exhibit varied physical and chemical properties that can be utilized in the fabrication of diverse functional materials. For instance, nanoparticles and microspheres are suitable for drug delivery systems although fibers and films are ideal for medical dressings and tissue repair materials [49]. Concerning shape, FPBHs can be tailored into various configurations including sheets, spheres, rods, etc., based on specific needs. Different shaped FPBHs possess unique application characteristics that make them suitable for preparing a wide range of medical dressings and repair materials. For example, flake-shaped FPBHs can be employed to locally wrap wounds whereas globular FPBHs are well-suited for cavity filling and repair [49].

The physical properties of FPBHs are diverse and adjustable, which can be regulated and optimized by adjusting preparation conditions and structural morphology, providing a wide range of possibilities for their application in the medical and food health field.

### Chemical properties: composition, content, and chemical structure

The chemical properties of FPBHs primarily encompass their composition, content, and chemical structure. The polysaccharide component of FPBHs plays a pivotal role in exerting their bioactivity. Polysaccharides are composed of monosaccharide molecules combined in varying proportions, making it crucial to analyze the monosaccharide composition within polysaccharide molecules. All studies except for 2 [15,24] analyzed or described the monosaccharide composition of the polysaccharides used, as presented in Table 1. In terms of composition, the polysaccharide content of FPBHs serves as a crucial indicator of their chemical properties. The polysaccharide content typically refers to the mass fraction of polysaccharide molecules within the FPBHs and can be determined through chemical analysis methods.

Additionally, the molecular weight of polysaccharides is also an important parameter (Table 1). The variation in polysaccharide content can influence the physical and chemical properties, as well as application effects of FPBHs. For instance, high-content FPBHs exhibit increased viscosity and strength; however, they may also display compromised rheological properties. Regarding chemical structure, it encompasses the monomer composition of polysaccharide molecules, the bonding mode between monomers, and their spatial arrangement. Distinct polysaccharide molecules possess diverse chemical structures that contribute to disparities in the properties and application effects of FPBHs.

The chemical properties of FPBHs are determined by multiple factors including their composition, content, and chemical structure. A comprehensive understanding of these chemical properties facilitates a better comprehension of their physical, chemical, and biological characteristics although offering novel possibilities for applications in medical and health domains.

### Functional attributes: rheology, water absorption, and stability

The functional characteristics of FPBHs encompass rheological properties, water absorption, and stability. FPBHs exhibit favorable rheological properties and can undergo stress-induced rheological changes to form a soft gel state, which is attributed to their physical properties. This rheological property can be modulated by adjusting factors such as polysaccharide molecule concentration, temperature, and pH value to meet diverse application requirements. For instance, in the preparation of medical dressings, the rheological properties of FPBHs can be tailored for enhanced adherence to wound surfaces and improved promotion of wound healing [50].

Moreover, FPBHs possess excellent water absorption capabilities that enable rapid swelling and formation of stable gel states. This water absorption property renders FPBHs highly versatile in applications within the medical and health fields including drug-containing hydrogel formulation and medical dressing development. For instance, in the formulation of drug release systems, the water absorption capacity of FPBHs can be harnessed to encapsulate drugs within hydrogels and achieve controlled drug release effects [51]. FPBHs exhibit excellent stability, maintaining their gel state and chemical properties unaltered for a specific duration. This inherent stability ensures the reliability and safety of FPBHs in medical material preparation. For example, FPBHs can be employed in fabricating medical dressings, with their stability guaranteeing no rupture or drug leakage during usage [50].

The functional attributes encompass rheological properties, water absorption capability, and stability; collectively rendering FPBHs highly promising for diverse applications within the realm of medicine and healthcare.

### Biomedical applications of food-derived polysaccharide-based hydrogels

A total of 32 studies [[9], [10], [11], [12], [13], [14], [15], [16], [17], [18], [19], [20], [21], [22], [23], [24], [25], [26], [27], [28], [29], [30], [31], [32], [33], [34], [35], [36], [37], [38], [39], [40]] investigating the application of specific food-derived functional polysaccharides in biomedical research were included, all of which encompassed *in vitro* experiments (Table 2). Among these, 21 studies [9,[12], [13], [14],^16^,^17^,^19^,[21], [22], [23],[28], [29], [30], [31], [32], [33], [34], [35],[38], [39], [40]] also incorporated *in vivo* experiments conducted on animal models (Table 3). The review encompasses an analysis of the biomedical effects observed from these studies along with a comprehensive evaluation of polysaccharide sources and composition as well as other ingredients utilized. Additionally, the research models employed and biological responses elicited have been thoroughly examined. The potential utilization of functional polysaccharides derived from food sources in human applications is illustrated in Figure 4.

**Table 3:** Biological effects of FPBHs in animal experiments.

Effect	Polysaccharide	Model	Biological responses	Reference
Effect of promoting osteogenesis	BSP	A murine dorsal subcutaneous pocket model (human MSCs and strain C57BL/6 mouse MSCs)	The MSCs-laden hydrogels in a murine dorsal subcutaneous pocket model demonstrated efficient macrophage activation, desirable scaffold-tissue integration and improved osteogenic differentiation in the delivered cells.	[9]
Efficacy in promoting wound healing	BSP	KM mouse full-thickness wound model (diameter ∼10mm)	The BSP/CMC/CBM_940_ hydrogel promoted neovascularization and accelerated wound healing.	[12]
Efficacy in promoting wound healing	ASP	Carrageenan-induced foot edema model; rat full-thickness wound model (1.5×1.5 m^2^)	The PVA-AP hydrogel effectively improved foot edema through anti-inflammatory effect and promote wound healing in rats.	[13]
Biocompatibility	TKP	Acute dermal skin erosion test in SD rats, and BALB/c mice were injected subcutaneously	The TKP-AA hydrogel did not cause any skin irritation or corrosion in SD rats. Injection of the hydrogel did not produce any toxicity to the organs of the mice.	[14]
Efficacy in promoting wound healing	KGM	SD rats defect model with 8 mm diameter full-thickness on the back of	P-OK as a wound dressing can effectively shorten the inflammatory period of wound and promote wound repair.	[16]
Efficacy in promoting wound healing	BSP	Female ICR mice a full-layer wound (diameter ∼8 mm) was prepared on the back	The hydrogels enhanced wound healing by inducing epidermal thickening and facilitating angiogenesis.	[17]
Efficacy in promoting wound healing	BSP	Rattus norvegicus rat open wound model (diameter 3 mm)	This hydrogel allowed rapid wound healing, restoring the skin wound structure to the full thickness in the body.	[19]
Antitumous effect	AP	C57BL/6 mouse tumor transplantation model with subcutaneous injection of B16-F10 cells	This hydrogel inhibited subcutaneous melanoma growth in mice	[21]
Efficacy in promoting wound healing	Paramylon	SD rats with subcutaneous injection and implantation on their back; Acute wound repair: a model of full-skin defects in rats back; Chronic wound repair: a third-degree scald model on the rat back	The paramylon hydrogel could reduce wound inflammation and promote angiogenesis to facilitate wound repair; it could promote the formation of blood vessels via the HIF-1α-VEGF pathway.	[22]
Efficacy in promoting wound healing Hemostatic effect	BSP	C57BL/6 mouse Liver hemorrhaging model; 6 mm full-thickness skin wounds in high-fat feed and streptozotocin induced male C57BL/6 mice diabetic models; 8 mm diameter full-thickness skin in ICR mice	The B-G hydrogel could accelerate angiogenesis and inhibit the progression of hepatic hemorrhage. It can boost wound closure by normalizing epidermal tissue regeneration and depositing collagen appropriately.	[23]
Hemostatic function	BSP	KM mice were used in liver and tail amputation hemorrhage models	The wound sealing and hemostatic properties of hydrogel *in vivo* were further evaluated using rat and mouse liver hemorrhage and rat tail amputation models.	[27]
Efficacy in promoting wound healing	LP and MP	A BALB/C mouse model of a full-thickness skin wound (6 mm deep and 2 mm diameter)	OPHs hydrogel was able to promote skin wound healing by improving the recovery ability of collagen cells.	[28]
Hemostatic effect	BSP	SD rat liver hemorrhage model	The COF hydrogel could inhibit the progression of hepatic hemorrhage, achieving hemostasis within 30 s.	[29]
Antibiosis Anti-inflammatory effect Efficacy in promoting wound healing	ABP	Bacterial infection wound model in rats with full-thickness skin wounds on the back inoculated with *S. aureus.*	The AP/Honey@PVA hydrogels could promote infected wound healing by regulating inflammation, promoting collagen deposition, growth factor production, and angiogenesis.	[30]
Antitumous effect Anti-inflammatory effect Antibiosis Efficacy in promoting wound healing	FU	B16-F10 cells were injected subcutaneously into the back of KM mice to construct tumor models *in situ*; a full-layer skin defect wound model (An 8 mm round full-layer defect was established on the back of KM mice) co-infected by *S. aureus* and *E. coli*	The CMC/OF/AuNPs@TA hydrogel effectively enhances wound healing following melanoma resection and impedes the infiltration of melanoma cells into cutaneous wounds. Moreover, hydrogels expedite wound healing by suppressing bacterial proliferation, attenuating the expression of inflammatory mediators in infected wounds, and promoting angiogenesis.	[31]
Anti-inflammatory effect	FU	Puncture induced disc herniation in SD rat model	By promoting the polarization of macrophages toward M2 phenotype and modulating the inflammatory response in rats, the DexMA-FU hydrogel effectively enhanced collagen reconstruction at the lesion site, thereby facilitating intervertebral disc repair.	[32]
Efficacy in promoting wound healing Hemostatic effect	BSP	Rat bleeding liver model and mouse bleeding tail amputation model; Model of full-skin defect on the back of SD rats (round, diameter 10mm)	The hydrogel exhibits the ability to expedite blood coagulation at the site of bleeding in mice and facilitate platelet aggregation for achieving hemostasis. Furthermore, it demonstrates potential to reduce inflammation and accelerate skin healing at the wound site through the promotion of angiogenesis factor (CD31).	[33]
Effect of promoting osteogenesis	FU	New Zealand white rabbit model with full-layer cartilage defect	The Nap-FFGRGD/FU hydrogel exhibits the potential to enhance oxidative stress (SOD, CCL4, CCL20) and mitochondrial function via the Nrf2 pathway, thereby facilitating cartilage regeneration in rabbits with full-layer cartilage injury.	[34]
Anti-inflammatory effect Efficacy in promoting wound healing	FU	Traumatic ulcers of wounds ∼5 mm in diameter on the cheek of SD rats	The hydrogels facilitate angiogenesis, mitigate the activity of pathogenic bacteria, prevent infection on ulcer surfaces, and attenuate neutrophil adhesion-mediated inflammatory responses to promote wound healing.	[35]
Efficacy in promoting wound healing Hemostatic effect	OP	Liver hemorrhage in mice model; the rat full-thickness skin defect model (4 round full-thickness skin wounds with a diameter of 1 cm were made on the skin with a hole punch)	The XG/OP hydrogel has the potential to expedite wound healing through its ability to enhance cellular proliferation, facilitate angiogenesis and hemostasis at the site of injury, as well as mitigate inflammatory oxidative stress levels.	[38]
Efficacy in promoting wound healing	BSP	SD rat wound model with a diameter of 10 mm was created on the back	The hydrogels play a role in the later stages of wound healing by being anti-inflammatory, pro-vascularization, and epithelialization, ultimately speeding up the wound healing process.	[39]

ABP, *Aloe barbadenis* polysaccharide; AP, *Astragalus* polysaccharides; ASP, *Avena sativa L.* polysaccharides; BSP, *Bletilla striata* polysaccharide; FU, fucoidan; KGM, Konjac glucomannan; LP, *L. japonica Thunb.* polysaccharide; MP, *M. haplocalyx Briq.* polysaccharide; MSCs, mesenchymal stem cells; OP, Okra polysaccharide; *S. aureus*, *Staphylococcus aureus*; TKP, *Tamarind Kernel* polysaccharide.

### Biocompatibility of FPBHs: potential benefits for medical and nutritional purposes

The biocompatibility of FPBHs serves as a crucial foundation for their application in the fields of medicine and biological sciences. Biomaterials exhibiting excellent biocompatibility can be readily accepted by human tissues without eliciting noticeable immune rejection or toxic reactions, although also possessing the potential to interact with surrounding tissues, thereby facilitating tissue repair and regeneration. Twenty seven studies [[9], [10], [11], [12],[14], [15], [16], [17], [18], [19], [20],^23^,[26], [27], [28], [29], [30], [31], [32], [33], [34], [35], [36], [37], [38], [39], [40]] utilized cell models as research subjects to investigate the biocompatibility of FPBHs. The cell lines employed included macrophages THP-1 [9], RAW264.7 [10,11,15,16,20,22,23], hepatocytes HepG2 [26], fibroblasts L929 [9,19,27,52,[29], [30], [31],^33^,^35^,[38], [39], [40]] and NIH/3T3 [14, 16, 17, 23, 30], human umbilical vein endothelial cells (HUVECs) [10,38], human embryonic kidney M293T [12], and mouse forestomach carcinoma cells (MFCs) [28], human keratinocyte cell HaCat [14], mouse neuroblastoma F11 [10,11,14], Saos-2 cells [10, 11], human mesenchymal stem cells (MSCs) [9,37], mouse MSCs [10, 11], mouse and rat bone-marrow derived preosteoclasts (BMMs) [10,11], and rat calvarial osteoblasts (RCOs) [10], MC3T3-E1 osteoblasts line [18], nasopharyngeal carcinoma cells (NPCs) [32], primary rabbit articular chondrocytes [34], mouse islet β cells (MIN6) [36].

Ten studies [11,12,14,15,19,23,26,28,29,32,34] suggested that FPBHs could enhance cell growth and proliferation. In 10 studies [12,17,19,27,29,31,33,[37], [38], [39]], fresh animal blood was employed to assess the hemolysis rate on hydrogels, revealing a hemolysis rate of <5%, thereby demonstrating favorable blood compatibility of FBPHs. The results indicated that FPBHs exhibited favorable biocompatibility with these cells without causing significant toxicity. The biocompatibility of FBPHs in animals was investigated in 1 study [14]. Choudhury et al. [14] developed a hydrogel composed of TKP and AA, which was applied in an acute dermal skin erosion model in SD rats, demonstrating no exacerbation of wound damage; additionally, subcutaneous injection of FPBHs in BALB/c mice did not exhibit any toxic effects.

It can be seen that research on biocompatibility should explore the interplay between materials and organisms rather than solely observing the presence or absence of toxicity in interventions. Such research would have greater theoretical significance. FPBHs have potential value in tissue engineering. These studies serve as a foundation for the development of FPBHs for clinical use.

### Drug delivery systems of FPBHs for tumor treatment

**FIGURE 5:**
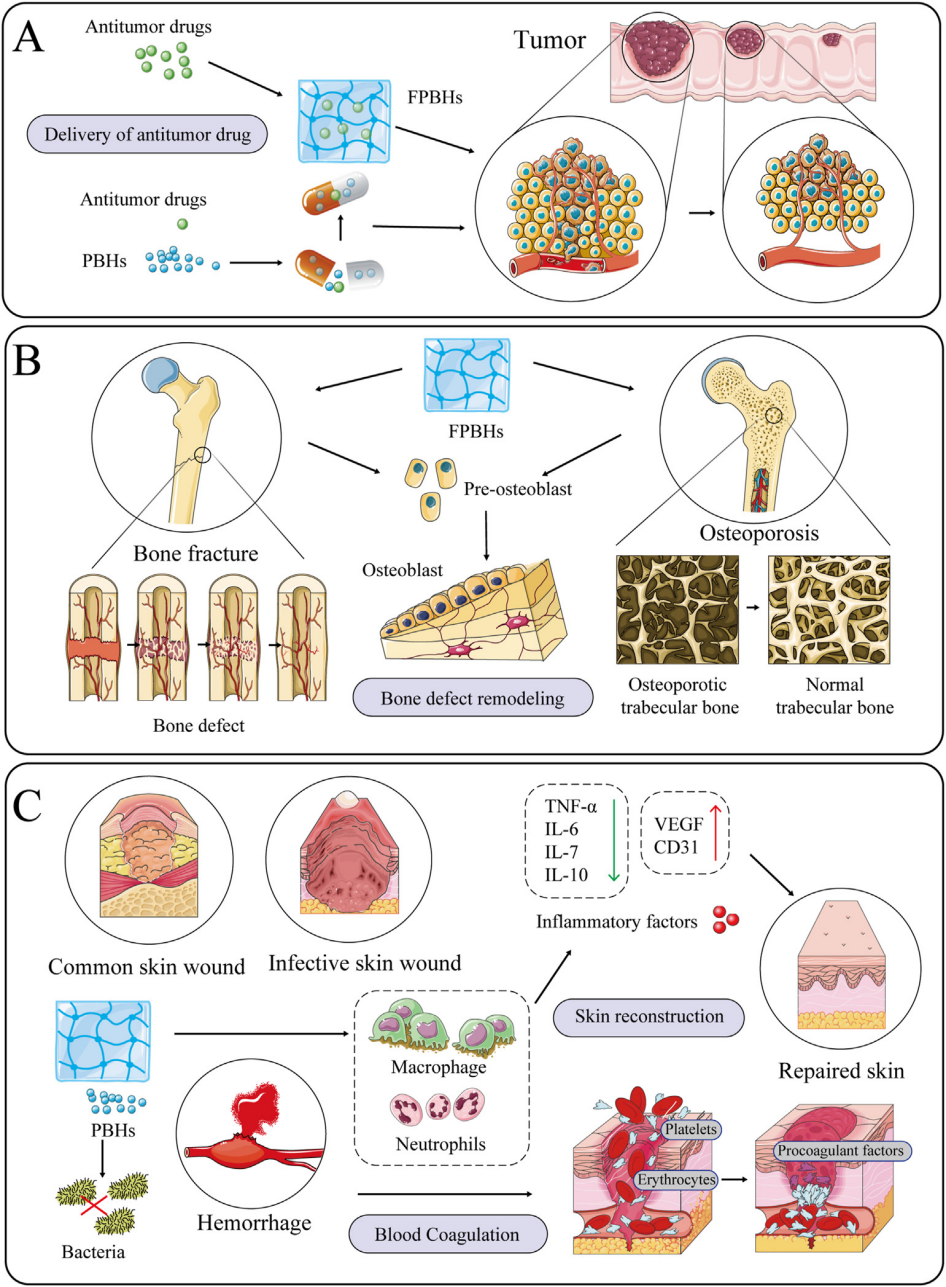
The application of food-derived polysaccharide-based hydrogels (FPBHs) as treatment strategies. (A) FPBHs inhibit the growth and proliferation of tumor cells by delivering and releasing anti-tumor drugs. (B) FPBHs alleviates bone injury or osteoporosis by promoting osteoblast differentiation. (C) Hydrogels treat skin wounds by being antibacterial, anti-inflammatory, and hemostatic.

The anti-tumor function of FPBHs is primarily manifested in 4 aspects: modulation of the tumor microenvironment, induction of tumor cell apoptosis, inhibition of tumor angiogenesis, and enhancement of immune function. Due to stringent screening criteria, only 3 studies [21, 25, 31] were included as a consequence of limited research on hydrogels directly derived from polysaccharides sourced from food origins. In addition to their inherent anti-tumor activity, hydrogels are frequently employed as carriers for encapsulating and delivering anti-tumor drugs in the treatment of neoplasms (Figure 5A**)**.

The anti-tumor efficacy of FPBHs was demonstrated *in vitro* using mouse melanoma cell line B16-F10 [21, 31], human HeLa cells [25], and mouse 3T3-L1 cells [25] *in vitro*. Kang et al. [21] embedded Apatinib in hydrogels made of *Astragalus* and HA and found that it effectively suppressed the growth and reproduction of Mouse melanoma cell B16-F10 cell line; then, they injected B16-F10 cells subcutaneously into C57BL/6 mice, and used FPBHs for intervention. It was found that FPBHs could effectively inhibit vasculogenic mimicry formation and melanoma growth in a mouse tumor transplantation model. The CMC/OF/AuNPs@TA hydrogel was synthesized by Chang et al. [31] using oxidized FU, tannic acid (TA) modified gold nanoparticles and CMC. The ACMC/OF/AuNPs@TA hydrogel demonstrated significant inhibition of tumor B16-F10 cell growth and proliferation *in vitro*, and when injected around the tumor of KM mice injected with B16-F10 cells on their back, effectively suppressed tumor expansion. Nagaraja et al. [25] reported the development of functionalized SPL seeds polysaccharide-based hydrogels, which serve as an effective carrier for targeted delivery of 5-Fluorouracil (5-FU) and exhibit potent inhibitory effects on the growth and proliferation of human HeLa and mouse 3T3-L1 cells, although also showing increased DPPH scavenging activity. The dose-dependent cytotoxicity of various hydrogels against HeLa and 3T3-L1 cells. The results unequivocally demonstrate the anticancer efficacy of the 5-FU-loaded hydrogel, exhibiting a reduction in activity by a factor of 2.5 and two-fold compared to pure 5-FU. It is anticipated that the release of 5-FU from the hydrogel networks is delayed.

The presence of FPBHs exerts a significant impact on the oxygen and nutrient supply in the vicinity of tumor cells, thereby creating an unfavorable milieu for tumor cell proliferation, consequently modulating the tumor microenvironment and impeding both tumor growth and metastasis. The Fucoidan-Manganese Dioxide Nanoparticles developed by Sung-Won et al. [53] exhibit the potential to downregulate the expression of phosphorylated vascular endothelial growth factor receptor 2 (VEGFR2) and CD31 in tumors, thereby demonstrating their ability to inhibit tumor angiogenesis. The inhibitory effect can be ascribed to the biological activity of fucoidan incorporated within the materials. The immunobiological activity of the polysaccharide is also the foundation of the anti-tumor ability of the hydrogel. By promoting T cell activation and proliferation, polysaccharide can enhance immune surveillance and improve the clearance of tumors, such as *Codium fragile* [54] and *Angelica dahurica* [55] polysaccharides, so they can be made into hydrogels to achieve the same or better effect. FPBHs have the ability to activate multiple apoptotic pathways, including the mitochondrial and death receptor pathways, leading to the induction of tumor cell apoptosis and exhibiting anti-tumor properties [56,57]. However, hydrogels exhibiting such characteristics may pose challenges in terms of biocompatibility and potential induction of apoptosis in normal cells.

A pivotal breakthrough in this domain lies in the ability to precisely target specific tumors although demonstrating tailored anti-tumor functionalities, necessitating further advancements in hydrogel design to enhance specificity and biocompatibility.

### FPBHs promote osteogenesis to repair bone defects and bone loss

Polysaccharide molecules has a good osteogenic function, which can promote the proliferation and differentiation of bone cells, as well as the formation and growth of new bone tissue [58]. The hydrogels composed of polysaccharide molecules with this capability can serve as both scaffolds in bone tissue engineering and therapeutic agents for bone injury repair. The treatment of fractures and osteoporosis involves the injection of hydrogels into the targeted site, wherein the active factors within stimulate preosteoblasts to differentiate into osteoblasts, thereby enhancing bone trabecular density for therapeutic purposes (Figure 5B).

Six studies [[9], [10], [11],^18^,^34^, ^37^] have found that FPBHs could promote the expression of osteogenic genes in different cells, and may have some benefits in repairing bone damage. Two studies [10,11] demonstrated that the surface of the *Tamarind kernel* powder olephan-acid (TKP-AA) hydrogel increased mouse and rat BMMs and mouse MSCs in the absence of osteogenic inducers, although also enhancing the expression of osteogenic genes in Saos-2 cells; it suggested a potential benefit for promoting osteogenic differentiation of cells. Halperin-Sternfeld et al. [18] found that MC3T3-E1 osteoblasts had increased calcium deposition on red marine microalga Porphyridium sp. Polysaccharide complex hydrogel, and had certain osteogenic ability. MC3T3-E1 preosteoblasts exhibited higher cell viability on the hydrogel compared to the control group, indicating favorable biological activity of FmocFF/PS hydrogel. Furthermore, significant calcium deposition on FmocFF/PS hydrogels suggests excellent osteogenic potential. The Nap-FFGRGD/FU hydrogel was synthesized by Zhao et al. [34] through the cross-linking of FU and Nap-FFGRGD peptide via a simple physical mixing method. This hydrogel can serve as a culture substrate to effectively enhance the osteogenic differentiation of primary rabbit articular chondrocytes. Similarly, Egle et al.’s hydrogels made from FU and chitosan also promote osteogenic differentiation of human MSCs derived from bone marrow [37].

Two animal studies utilized C57BL/6 mice as a model, where human MSCs coated hydrogel was subcutaneously injected [9], and the hydrogel was externally applied to full-layer bone defect wounds in New Zealand rabbits [34]. Niu et al. [9] reported that acBSP, a hydrogel prepared by BSP, could promote The expression of pro-osteogenic/-angiogenic in humans monocyte THP-1 cell line genes. The acBSP not only facilitates a tightly integrated surface with an ample number of tissue cells but also establishes a porous polysaccharide network that effectively enhances the proliferation of host tissue cells on days 14 and 28. Moreover, subcutaneous injection of acBSP-loaded human or mouse mesenchymal stem cells into the dorsal region of mice demonstrated that hydrogels functioned as effective scaffolds for tissue integration by inducing macrophage activation and enhancing bone differentiation *in vivo*. The Nap-FFGRGD/FU hydrogel, when applied to the bone defect, exhibited remarkable efficacy in promoting cartilage regeneration in rabbits with full-layer cartilage injury by augmenting oxidative stress (SOD, CCL4, CCL20) and mitochondrial function through activation of the Nrf2 pathway [34].

Polysaccharide molecules within the hydrogel can activate cellular signal transduction pathways by binding to cell surface receptors, thereby promoting bone cell proliferation and differentiation, as well as accelerating bone tissue repair and regeneration [58]. Furthermore, these polysaccharide molecules can interact with the extracellular matrix to form a scaffold structure that provides support and protection for newly generated bone cells, facilitating bone tissue growth and repair. Additionally, polysaccharides are capable of regulating the expression and release of growth factors and immune factors, further promoting bone tissue growth and repair [58]. Polysaccharides have the ability to bind and stabilize growth factors, thereby extending their half-life, augmenting their biological activity, facilitating the proliferation and differentiation of bone cells, as well as promoting the formation and growth of new bone tissue [59].

Therefore, FPBHs exhibits extensive potential in the realm of bone tissue engineering and repair, rendering it an exceptional biomaterial for addressing bone defects, promoting bone healing post-osteotomy, facilitating bone transplantation, and other related applications.

### Topical application efficacy of FPBHs in promoting skin wound repair

FPBHs, possessing remarkable bioactivity and histocompatibility, exhibit promising potential as a material for facilitating wound healing. Polysaccharide molecules in the hydrogel can bind to receptors on the surface of cells, promote cell proliferation, differentiation, and migration, thereby forming new tissue and repairing damaged tissue [60]. FPBHs can contribute to the skin regeneration of both common and infected wounds by exerting bacteriostatic, immunomodulatory, and hemostatic effects (Figure 5C).

Fourteen studies [12,13,16,17,19,22,23,28,30,31,33,35,38,39] have reported that FPBHs could accelerate the healing of animal wounds through external application on the basis of their biocompatibility. The BSP/carboxymethyl chitosan/Carbomer940 (BSP/CMC/CBM_940_) hydrogel developed by Huang et al. [12] demonstrated accelerated wound healing in a Kunming mouse full-thickness wound model, with a diameter of ∼10mm, compared to the control group without FPBHs. the oxidized plant-polysaccharides-chitosan hydrogel (OPHs), as demonstrated by Xue et al. [28], exhibited the potential to enhance skin wound healing in a BALB/C mouse full-thickness skin wound model (6 mm deep and 2 mm diameter) through augmentation of collagen cell recovery ability. Jakfar et al. [19] also used BSP to make methylcellulose/ BSP (MB) hydrogels, apply it to the model, and found that it can recover the wound faster and reconstruct the skin tissue more completely.

In another study [22], 2 models were created using SD rats, namely an acute wound model (representing full-skin defects on the rat back) and a chronic wound model (representing third-degree scalds on the rat back). By evaluating the topical application of FPBHs in these models, researchers observed that paramylon hydrogel facilitated recovery of both acute and chronic wounds by promoting vascular regeneration through HIF-1α-VEGF signaling although reducing local inflammatory reactions. Similarly, Zhu et al. [16] utilized P-OK hydrogel (an injectable hydrogel by mixing the adipic acid dihydrazide modified γ-polyglutamic acid with oxidized Konjac glucomannan) in a full-thickness defect model with 8 mm diameter on the back of SD rats and observed that P-OK as a wound dressing effectively shortened the inflammatory period of wounds and promoted wound repair. El Hosary et al. [13] employed carrageenan-induced foot edema model and rat full-thickness wound model (1.5×1.5 m^2^) to validate the efficacy of polyvinyl alcohol / Avena sativa L. polysaccharides (PVA/ASP) hydrogel in promoting wound healing.

FPBHs exhibits favorable moisturizing and permeability properties, thereby effectively preventing wound desiccation and bacterial infection although facilitating wound repair and healing [61]. Furthermore, the regulation of growth factor expression and release by FPBHs can promote wound healing. The incorporation of polysaccharide molecules within the hydrogel enables binding and stabilization of growth factors, leading to prolonged half-life and enhanced biological activity. Consequently, this promotes cellular proliferation, differentiation, migration, as well as expedites wound healing and repair processes [61].

Anti-inflammatory effect of FPBHs through immune response

Polysaccharide molecules in the hydrogel might bind to receptors on the surface of immune cells, activate the function of immune cells, thereby regulating the function of the immune system and inhibiting the occurrence of inflammatory reactions [62,63]. Taking skin wounds as an example (Figure 5C), FPBHs can facilitate tissue repair by stimulating immune cells such as macrophages and neutrophils, although also regulating the expression of inflammatory factors like IL-6, IL-7, and TNF-α.

Six articles [15,22,23,30,31,35] indicated that FPBHs exhibit significant efficacy in reducing inflammation Three studies [15,22,23] demonstrated the impact of FPBHs on immune response in RAW264.7 cells, although another study, one study [30] revealed their potential to mitigate inflammation by modulating the expression of immune factors in infected wounds in rats. Liu et al. [23] fabricated a composite material consisting of BSP, gelatin, and methyl acrylate using ultraviolet light cross-linking technique to prepare PHBs, which was found to effectively induce the polarization of RAW264.7 macrophages toward an anti-inflammatory phenotype. The CMC/OF/AuNPs@TA hydrogel, prepared by Chang et al. [31], was applied to the surgical wound following melanoma resection in mice; remarkably, this hydrogel not only failed to accelerate tumor growth but also effectively mitigated the inflammatory response of the wound, thereby promoting wound healing. The CA/FU hydrogel loaded with *Lactobacillus rhamnosus*, as prepared by Dou et al. [35], was applied to treat traumatic ulcers of wounds ∼5 mm in diameter on the cheek of SD rats. This intervention effectively mitigated neutrophil adhesion-mediated inflammatory responses.

In 2 other studies, Soy protein isolate -*Flammulina velutipes* polysaccharide (SPI-FVP) hydrogel is able to isolate the protein by promoting the expression of IL-6, IL-10 and TNF-α for inhibiting the pro-inflammatory effects of RAW264.7 cells [15], although Paramylon hydrogel inhibited the inflammatory response of LPD-induced macrophages by reducing the levels of TNF-α and IL-7 [22]. In immunohistochemical experiments, Zhang et al. [30] found that AP (*Aloe barbadenis* polysaccharide) / Honey @ PVA hydrogels can significantly reduce the expression of IL-6 and TNF-α in rat infected wounds, promote the expression of VEGF and CD31, thus regulating inflammatory response and collagen deposition.

The anti-inflammatory activity of FPBHs may be related to the anti-inflammatory activity of polysaccharide molecules in hydrogels. Among them, the activation of T cells by polysaccharide molecules is the most prominent. Polysaccharide molecules in the hydrogel can bind to receptors on the surface of T cells, thereby activating their functions [64]. This activation effect includes increasing the proliferation of T cells, promoting the differentiation of T cells, and regulating the immune activity of T cells, thereby enhancing the immune system [65]. Polysaccharide molecules can also activate the functions of macrophages and dendritic cells [66]. Macrophages and dendritic cells are important immune cells in the immune system.PBHs can interact with these immune cells, thereby stimulating them to release various cytokines and signaling molecules, enhancing their immune function, and thus enhancing the clearance ability of the entire immune system [67]. Polysaccharide molecules can not only regulate immune cells, but also bind to inflammatory mediators to neutralize the effect of inflammatory mediators and reduce the severity of inflammatory response [68]. Besides, polysaccharide molecules also exert anti-inflammatory effects by regulating the occurrence of oxidative stress reactions [69]. They can inhibit the occurrence of oxidative stress reactions, reduce the production of oxygen free radicals, thereby reducing the severity of inflammatory reactions.

Therefore, FPBHs has broad application prospects in the treatment of inflammatory diseases, promotion of wound healing, immune therapy and other aspects, and can be used as an ideal biomedical material.

### Inhibitory effect of FPBHs on microorganisms

FPBHs has a strong antibacterial effect, which can effectively inhibit the growth and reproduction of various bacteria and fungi [70]. Six studies [13,16,24,30,31,35], have found that FPBHs can inhibit the growth and reproduction of microorganisms *in vitro*, thereby playing a certain prevention and control of infection. Zhang et al. [30] found that AP/Honey@PVA hydrogel showed significant growth inchibition against *Staphyloccus aureus* (*S. aureus*), *Escherichia coli* (*E. coli*), and fungus *Candida albicans* (*C. albicans*). The AGNPS Colloids hydrogel produced by Massironi et al. [24] has inhibitory effects on Gram-negative (*E. coli* and *Pseudomonas aeruginosa*) and Gram -positive (*S. aureus*) bacteria. Similarly, the P-OK hydrogel made by Zhu et al. [16] and the CMC/OF/AuNPs@TA hydrogel made by Chang et al. [31] indicated their inhibitory action for *S. aureus* and *E. coli*; and El Hosary et al. produced PVA-AP Hydrogel to effectively control bacterial reproduction, including gram-positive (*S. aureus* and *Micrococcus Leutus*) and gram-negative (*E. coli* and *Pseudomonas Aeruginosa*) [13]. The CA/FU hydrogel loaded with *Lactobacillus rhamnosus* prepared by Dou et al. [35] had inhibitory effects on *S. aureus* and fungus *C. albicans.* However, the mechanism of hydrogels inhibiting microbial growth has not been deeply explored in these studies.

The inhibitory effect of FPBHs is primarily attributed to the interaction between polysaccharide molecules in the hydrogel and bacterial and fungal structures, including the cell wall, cell membrane, and other components. This interaction disrupts their normal metabolism and physiological functions, thereby impeding their growth and reproduction [70]. At present, the research on FPBHs antibacterial ability is mainly verified by *in vitro* experiments. *In vivo*, FPBHs enhance its antibacterial effect by regulating the function of the immune system. Polysaccharide molecules in the hydrogel bind to receptors on the surface of immune cells, activate the function of immune cells, enhance the clearance ability of the immune system, and strengthen the attack and clearance of bacteria, achieving the antibacterial effect [71]. Experiments conducted within living organisms predominantly rely on indirect indicators such as inflammation levels or infection markers; direct observation of microorganism proliferation is limited. Furthermore, research on fungi and viruses remains scarce.

Therefore, FPBHs hold significant potential for diverse applications in medical care settings where various antibacterial materials can be developed (e.g., mouthwash solutions or surgical supplies).

### Hemostatic effect of FPBHs on hepatic and tail hemorrhage

The hydrogel's polysaccharide molecules may elicit the activation of clotting factors within the bloodstream, thereby inducing local vasoconstriction or expediting platelet aggregation, thus exerting a role in hemostasis. Five studies [23,27,29,33,38] employed murine or rodent liver bleeding models, although 2 studies [27, 33] utilized the mouse tail amputation model to investigate the hemostatic properties of hydrogels.

The XG/OP hydrogel, prepared by Liu et al. [38], was injected into the liver wound of liver hemorrhage mice model, resulting in a significantly reduced bleeding volume (22.6±6.8 mg) compared to the normal control group (45.7±9.6 mg); this observation suggests that the hydrogel not only adhered to the wound site and facilitated physical hemostasis but also triggered platelet activation. The BG hydrogel prepared by Zhang et al. [33] exhibits a pronounced hemostatic effect in both rat bleeding liver and mouse bleeding amputation models. in rats model, the BG hydrogel group achieved rapid hemostasis within 32.2±1.8 s, although in the mouse model, it achieved rapid hemostasis within 325.6±53 s following amputation. The remarkable hemostatic efficacy of the BG hydrogel could be attributed to its microporous structure, which facilitates increased surface area for red blood cell adhesion and platelet aggregation. Furthermore, the presence of cationic gelatin with abundant positively charged amine groups contributes to its antihemorrhagic properties by promoting clot formation.

FPBHs has a significant hemostatic effect, which can quickly form clots and effectively control bleeding. Polysaccharide molecules in the hydrogel can bind to fibrinogen in the blood, promote fibrinogen aggregation and coagulation, and form fibrin clots, thereby achieving hemostasis. PBHs can also exert hemostatic effects by regulating platelet aggregation and activation. Polysaccharide molecules in the hydrogel can bind to receptors on the surface of platelets, promote platelet aggregation and activation, thereby enhancing the hemostatic effect. In addition, PBHs has good biocompatibility and biodegradability, which can be safely used in humans body without adverse effects.

FPBHs has broad application prospects in the fields of surgery, trauma, etc., and can be used to prepare various hemostatic dressings, hemostatic sponges, and other hemostatic materials, providing safer and more effective hemostatic measures for clinical medicine.

### PBHs facilitate insulin secretion of islet β cells

Given their excellent biocompatibility, FPBHs can serve as a favorable substrate for cellular growth and activity. Leveraging the unique biological properties of polysaccharide molecules, FPBHs have the potential to enhance the secretion of specific neuroendocrine cells. However, limited research has been conducted in this area, with only one study [36] reporting on the impact of hydrogel on mouse islet beta cell secretion.

Amin et al. [36] fabricated the PVA-FU-MA hydrogel through photocrosslinking, encapsulated MIN6 cell aggregates within the hydrogel, then conducted ATP detection to assess cell metabolic activity and glucose-stimulated insulin secretion (GSIS). The findings demonstrated that the intracellular ATP levels in the PVA-FU-MA hydrogel remained consistently elevated, although the GSIS test exhibited enhanced insulin secretion. Therefore, it can be concluded that the PVA-FU-MA hydrogel effectively promoted insulin secretion of MIN6 cells and serves as an excellent carrier for cell encapsulation. The secretory capacity of food-derived polysaccharides may be attributed to their biological activity, although further investigation is required to elucidate the specific underlying mechanism. If this unique characteristic of food-derived polysaccharides can be harnessed for hydrogel development, it holds immense potential in the realm of hormone secretion.

### Potential biomedical applications and nutritional value of FPBHs

In addition to their role in the biomedical field mentioned above, FPBHs may also hold potential applications in ophthalmology, as well as in the management of reproductive and metabolic chronic diseases.

Customizable hydrogels loaded with specific food-derived polysaccharides can exhibit tailored physical, chemical, and biological properties suitable for drug delivery carriers, tissue scaffolds, sealants, and injections. Injectable hydrogels utilizing the porous structure and biocompatibility have been investigated for corneal injury repair and regeneration of diseased corneal tissue in ophthalmology. Notably, a study demonstrated that a clear gelatin-based hydrogel containing hepatocyte growth factor significantly increased the thickness of regenerated epithelial cells on the gel after 5 days of culture using optical coherence tomography imaging assessment [72]. Furthermore, hydrogels hold great potential for treating other eye diseases such as glaucoma, retinopathy, eyelid abnormalities, and uveal melanoma. However, there is limited literature on specific applications of specific food-derived polysaccharides in ophthalmology despite their promising prospects.

The application of FPBHs in the field of reproduction is also applicable to female reproductive diseases, where hydrogels are expected to play a reparative role in endometrial injury, injured harem adhesion, follicle dysplasia, and low blastocyst formation rate. Particularly in the current focus on frozen eggs research, a study has demonstrated that FU can effectively mitigate ice crystal formation during cell freezing process, reduce mechanical damage to cells, and enhance cell survival rate and functional recovery post-thawing [73]. This component may hold potential value in embryo cryopreservation; however, further research is warranted for confirmation. In male reproductive diseases, hydrogels are anticipated to safeguard testicular tissue and stimulate testosterone production. The application potential of photo-crosslinked gelatin, alginate, and dextritol hydroxymethacrylate hydrogel was discussed for testicular tissue culture *in vitro*. Findings revealed that testicular tissue cultured on alginate methacrylate exhibited the highest tubule integrity rate at day 32 (0.835 ± 0.021), a high density of spermatocytes (2107.627 ± 232.082 /mm^2^), as well as a substantial proportion of SOX9-positive and well-preserved tubules at day 32 (0.473 ± 0.047) [74].

Due to the need for further verification of biomaterial safety, FPBHs have not been reported in clinical studies. Particularly for chronic metabolic diseases such as diabetes and its complications, metabolic fatty liver disease, atherosclerosis, and metabolic bone disease, long-term injection or oral intake of hydrogels would be required with higher safety requirements. However, although limited research has been conducted on FPBHs in chronic metabolic diseases, numerous studies have reported the beneficial effects of specific food-derived polysaccharides through oral treatment. For instance, Tibetan brassica polysaccharide alleviates hyperlipidemia in rodents [75] although Morchella polysaccharide reduces weight gain and colon inflammation caused by a high-fat diet in mice [76]. These examples demonstrate that FPBHs also hold potential for addressing chronic metabolic diseases. By customizing suitable material forms, biological activity-containing polysaccharide molecules can be delivered to target organs although improving the nutritional value of the polysaccharides themselves

### Conclusion and perspectives

Although FPBHs have wide application prospects in the medical and nutritional health field, there are still some difficulties in the research. First of all, the preparation and performance control of FPBHs still face certain challenges. Many factors should be considered in the preparation process of FPBHs, such as polysaccharide type, crosslinking degree, pH value, ion concentration, etc. These factors interact with each other in a complex way, which requires in-depth study to obtain high-quality FPBHs. In addition, the regulation of FPBHs also needs to be studied to meet different medical and nutritional needs. Second, the biological activity and biosafety of FPBHs still need to be further studied. At present, the metabolism, toxicity, and immunological effects of FPBHs *in vivo* are not enough, and further studies are needed. Finally, the large-scale production and application of FPBHs face certain challenges. The preparation of FPBHs requires the support of production technology and equipment. Besides, the application scenarios of FPBHs need to be further expanded and deepened to meet different medical and nutritional needs.

Despite the above research difficulties, FPBHs still have a broad application prospect in the medical and health fields. In the future, with the continuous progress of science and technology and in-depth research, it is believed that the application prospect of FPBHs will be more extensive and far-reaching. However, it is worth noting that although specific food-derived polysaccharides offer a promising avenue for the synthesis of FPBHs, research in this area is still in its infancy. The use of specific food-derived polysaccharide as a raw material for FPBHs has not yet been widely explored, and there is currently a lack of clinical data or retrospective studies summarizing the basic experiments conducted with these hydrogels. Therefore, further research is needed to explore the nutritional potential of natural polysaccharides in the synthesis of FPBHs, and to determine their effectiveness and safety for clinical applications.

## Data availability

Data described in the manuscript, code book, and analytic code will be made publicly and freely available without restriction at [URL]. For additional information please see our website at https://jn.nutrition.org/content/authorinfo/. ∗Please note, Data Sharing statements apply to Original Research Articles. Perspectives, Opinions, Editorials, and Letters to the Editor do not require this statement.

## Author contributions

Corresponding authors will be queried for their own and their co-authors’ conflict of interest disclosures during the manuscript submission process using the Declaration of Interests tool (https://declarations.elsevier.com/). The tool will generate a Word file for upload with your manuscript submission.

## Conflict of interest

There is no conflict of interest.
